# Early-Mid Pleistocene genetic differentiation and range expansions as exemplified by invasive Eurasian *Bunias orientalis* (Brassicaceae) indicates the Caucasus as key region

**DOI:** 10.1038/s41598-017-17085-8

**Published:** 2017-12-01

**Authors:** Marcus A. Koch, Florian Michling, Andrea Walther, Xiao-Chen Huang, Lisa Tewes, Caroline Müller

**Affiliations:** 1 Heidelberg University, Centre for Organismal Studies, Heidelberg, 69120 Germany; 2 Bielefeld University, Chemical Ecology, Bielefeld, 33615 Germany

## Abstract

Turkish Warty cabbage, *Bunias orientalis* L. (Brassicaceae) is a perennial herb known for its 250 years of invasion history into Europe and worldwide temperate regions. Putative centers of origin were debated to be located in Turkey, the Caucasus or Eastern Europe. Based on the genetic variation from the nuclear and plastid genomes, we identified two major gene pools in the Caucasian-Irano-Turanian region and close to the Northern Caucasus, respectively. These gene pools are old and started to diverge and expand approximately 930 kya in the Caucasus. Pleistocene glaciation and deglaciation cycles favoured later expansion of a European gene pool 230 kya, which was effectively separated from the Caucasian-Irano-Turanian gene pool. Although the European gene pool is genetically less diverse, it has largely served as source for colonization of Western and Northern Europe in modern times with rare observations of genetic contributions from the Caucasian-Irano-Turanian gene pool such as in North-East America. This study largely utilized herbarium material to take advantage of a biodiversity treasure trove providing biological material and also giving access to detailed collection information.

## Introduction

The evolutionary history of a species is a sum of complex spatio-temporal processes on very different scales often spanning several hundred thousand of years and whole continents. During the past 20 years, detailed phylogeographic studies accumulated aiming to resolve biogeographic patterns and evolutionary processes. These studies serve as important background information to understand and explore contemporary processes such as biological interactions^1^, species-environment interactions^2^, population differentiation and speciation^3^, or rapid adaptation and niche evolution^4^. Species and genus level phylogeographic analyses provided very surprising and unexpected results indicating, for example, multiple intercontinental dispersal^5^, unexpectedly fast colonization of large areas^6^, survival in glaciated landscapes^7^, or mosaic structure of species assemblages of different age of presumably old vegetation types^8^. Moreover, phylogeographic studies open an avenue to ask the question to what extent phylogenetic-phylogeographic history might have a major impact on later diversification and functional trait evolution. This phenomenon of constrained trait evolution by phylogenetic history has been introduced as phylogenetic inertia. Although the concept of phylogenetic inertia has changed during the history of evolutionary biology^9^ from ‘pattern’ definitions^10,11^ to ‘process’ definitions^12,13^, the concept generally remains important, and it is also reflected with a similar concept of phylogenetic niche shifts and conservatism^14^. A special case of rapidly changing patterns in space and time is given with invasive species. These species often colonize different continents within a few decades, thereby raising the question if phylogenetic constraints or rapid adaptation play a dominant role during such short time frames. There is little empirical evidence, and one large scale meta study focusing on the entire British flora came to the conclusion that the transition from non-invasive to invasive is not related to phylogenetic distinctiveness, but instead to environmental preferences^15^. Species invasiveness was correlated with higher nitrogen and moisture preference as abiotic traits highly associated with human-mediated disturbances. However, the situation might be different, when species become invasive in natural habitats and here, phylogenetic inertia, niche shifts and adaptation do play a major role.

**Figure 1:**
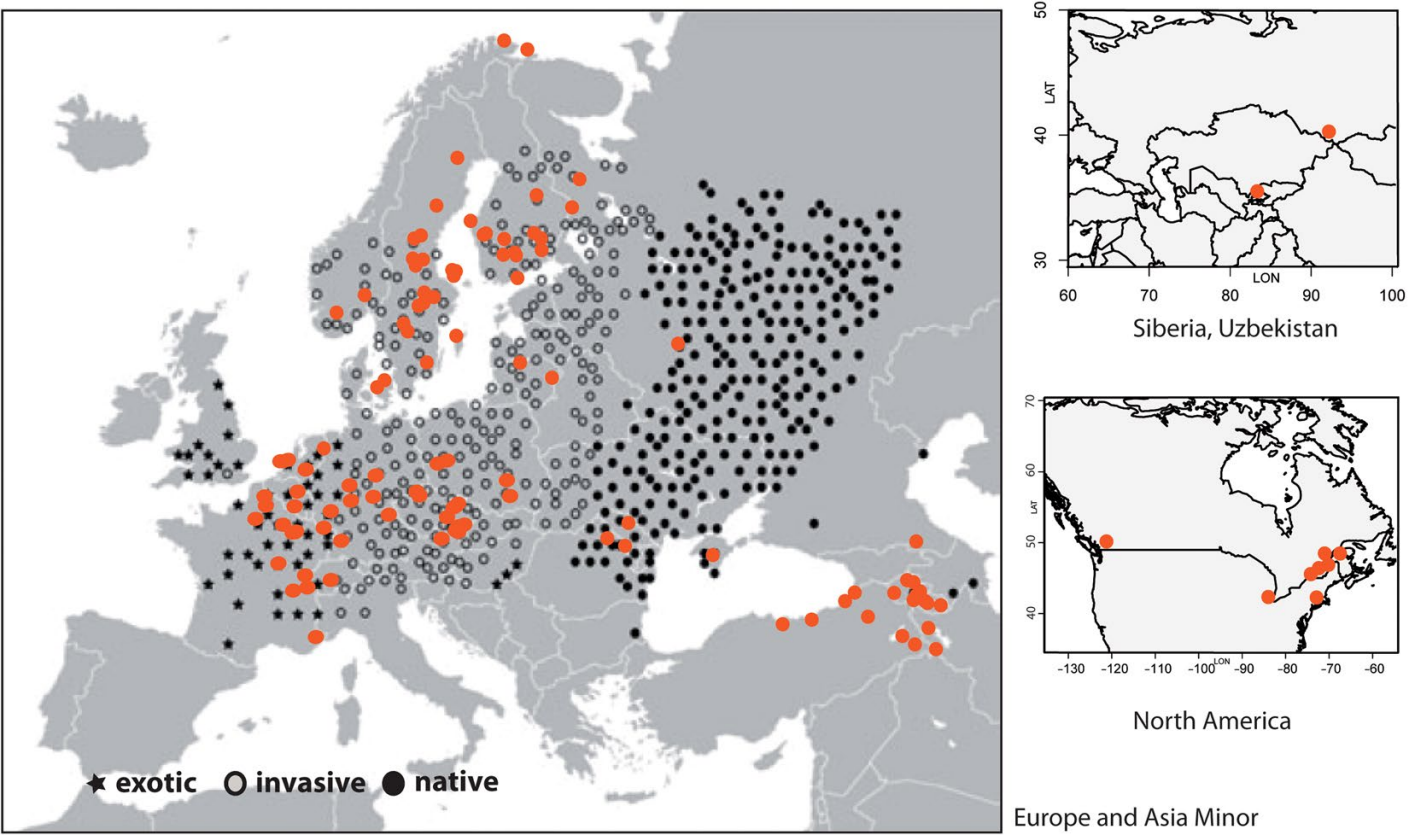
Distribution of sampled and analyzed accessions of *Bunias orientalis*. Previous classification of the species distribution range^16^ according to native, invasive (introduced during the last 250 years and invasive in this region) and exotic (recently introduced and not invasive) is indicated. Maps were generated in R using the mapdata package (A language and Environment for Statiscal Computing, R Core Team, R Foundation for Statiscal Computing, Vienna, Austria, 2017, https://www.R-project.org; mapdata: Extra Map Databases, R package version 2.2–6, https://CRAN.R-project.org/package=mapdata), and figures were drawn in Inkscape v 0.91 (https://inkscape.org/).

The Turkish Wartycabbage or Turkish Rockcress *Bunias orientalis* L. (Brassicaceae) is one of these invading species. The species has a primarily Eurasian distribution range, but its original distribution is not known. It was believed that the native range of *B*. *orientalis* is reaching from Eastern Europe to Central Asia including the Caucasus, the Irano-Turanian region and Central Asia^16^ (Fig. 1). It has been also hypothesized that *B*. *orientalis* is an indigenous floristic element of open grassland in mountainous and highland-(sub)alpine regions of the Southern Caucasus^17–19^. Interestingly, *B*. *orientalis* has only one sister species, *B*. *erucago* L., and there are no other members within the entire tribe Buniadeae, which is assumed to be older than 12 million years. This is a rare phenomenon in the entire family^20,21^ and might indicate only little evolutionary potential to diverge and adapt to new environmental conditions. However, during the last 250 years the species rapidly spread all over Central Europe and Scandinavia, throughout Asia towards China, and also reached the US and Canada several decades ago^22–24^. In most of these colonized regions, the long-lived perennial and self-compatible species turned out to be invasive, but no obvious shifts in its ecology could be detected^25^. Habitats in which the species is becoming invasive are often linear landscape elements such as grassland along rivers, rural sides along railways and field margins; but often the species is also able to colonize disturbed grassland.

Inconsistent results from few populations and RAPD and ISSR markers have been reported from Lithuania^26^, and a 9-year monitoring experiment in one population using also RAPDs showed that the population was able to maintain its genetic variation over time^27^. Thus, more detailed studies on its genetic variation are necessary.

Herein, we used whole plastid genome sequence data to provide a robust temporal framework for the phylogenetic history of *B*. *orientalis* over the last few millions of years. Furthermore, plastid DNA variation was screened on a large geographic scale to identify ancestral genepools and follow genetic footprints of this maternally inherited molecular marker, which is consequently indicating potential dispersal routes. Nuclear genome-wide polymorphisms were analyzed using amplified fragment length polymorphisms (AFLPs) to further confirm gene pools and detect potential admixture.

Unravelling a comprehensive evolutionary history of any species requires a representative sampling. Therefore, the present study was designed to take advantage of herbaria collections^28^ with their availability of material from otherwise hardly accessible areas and species’ distribution ranges spanning entire continents, and potentially important material from the recent past (decades) possibly highlighting introduction history. However, a drawback is that these collections most often provide single individuals per geographically defined population, and, therefore, an initial genetic screening on population level was conducted confirming that within population diversity can be ignored for the proposed questions to be unraveled.

In particular, we aimed to (1) unravel the putative center of origin of *B*. *orientalis*, to (2) identify the gene pool giving rise to invasive and spreading populations, and to (3) test whether range expansion is explained by bioclimatic niche shifts or genetic differentiation. This study should provide the spatio-temporal framework for future detailed experiments focusing on population-level processes^29,30^.

## Results

### Plastid genome analysis indicate Mid-Pleistocene divergence

**Figure 2:**
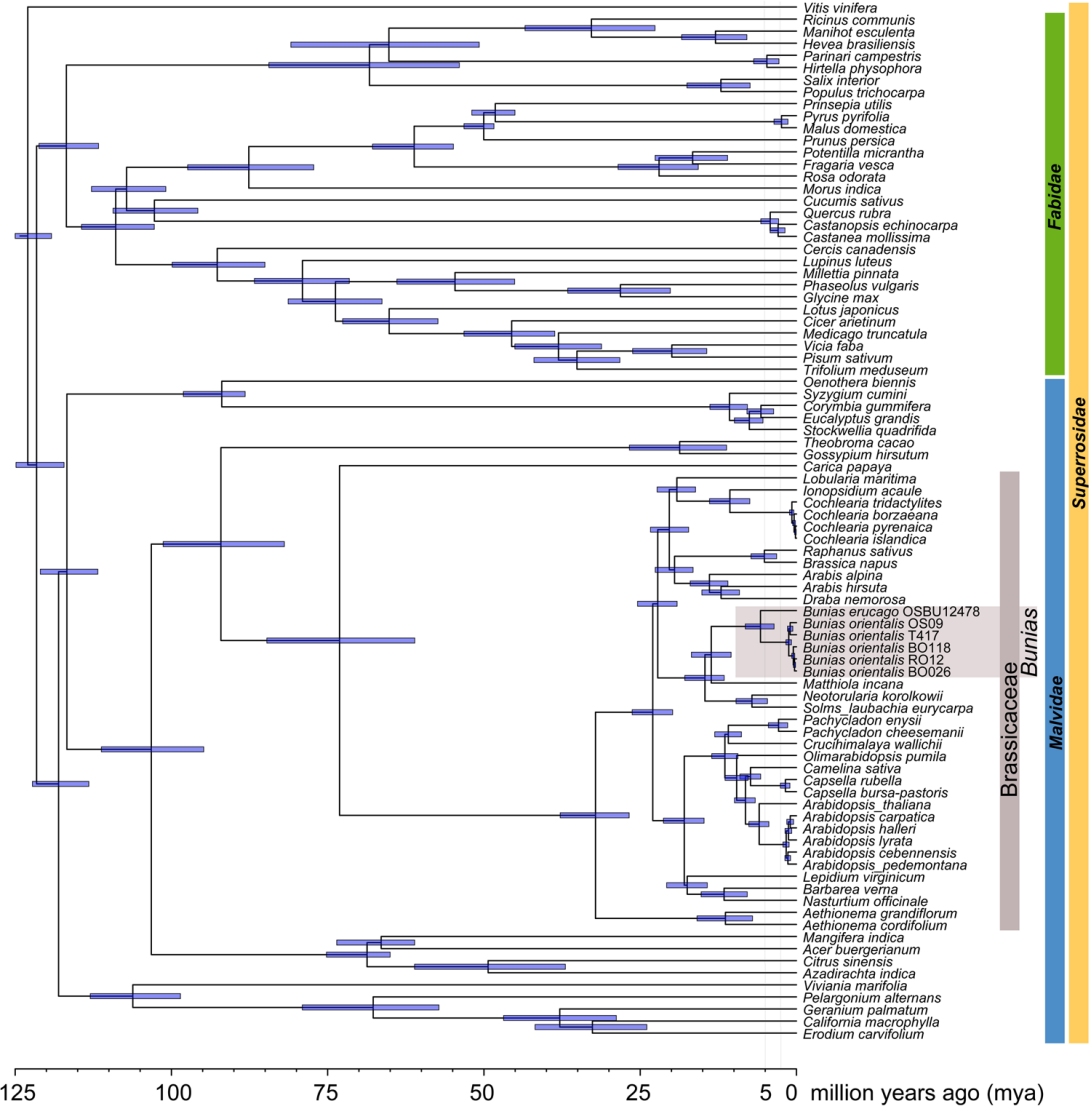
BEAST analysis of whole plastid genomes and divergence time estimates.

The fully assembled and annotated plastid genomes are databased with GenBank accession codes ENA LN877374-LN8773777. Phylogenetic analysis and divergence time analysis using BEAST were fully in agreement with our previous results^20^ and not discussed in detail herein. The final BEAST chronogram (Fig. 2) indicates a split time of 5.76 Mya (7.10–4.61 My; 95% confidence interval) between *B*. *erucago* and *B*. *orientalis*. *Bunias orientalis* plastid genomes refer to the following haplotype codes (as defined by the 3-gene-marker set, see below): T417 = H9, OS09 = H16, RO12 = H2, BO26 and BO118 = H6 (BO26 = H6a from Georgia and representing the Northernmost Caucasian accession analyzed, and BO118 = H6b from Poland). Within *B*. *orientalis* the split between European and Irano-Turanian-Caucasus haplotypes was dated with 1.23 Mya (1.89–0.56 My; 95% confidence interval). The split between the two selected Irano-Turanian-Caucasus haplotypes H9 and H16 was set to 0.99 Mya (1.65–0.32 My; 95% confidence interval). The split between the European (H2 from Romania) and the Northern Caucasus (H6a) was set to 0.30 Mya (0.49–0.11 My; 95% confidence interval). Both H6 haplotypes are similar to each other and are separated by 0.50 My of divergence, indicating the minimum age of this respective Caucasian subgroup. Using the divergence time between *B*. *erucago* and *B*. *orientalis*, the mean plastid genome-wide mutational rate is about μ = 7.798 × 10^−10^ mutations/site/year^20^. However, the mean rate obtained for the concatenated alignment of the three plastid sequences is faster and was calculated with μ = 2.004 × 10^−9^ mutations/site/year, which was used for further analyses.

### Plastid DNA haplotype assignment shows two gene pools

**Figure 3:**
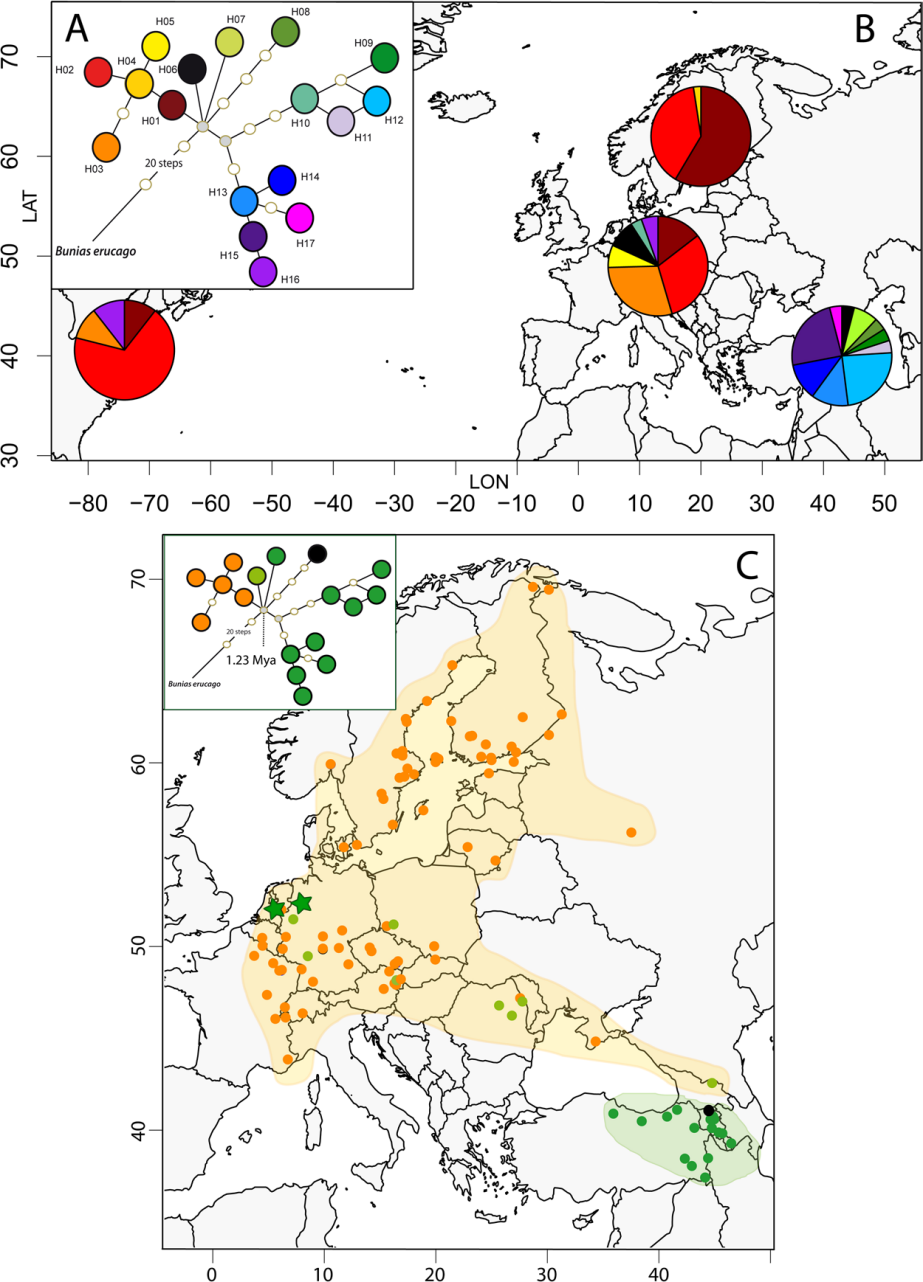
(**A**) Parsimony network of plastid haplotypes, (**B**) its frequency distribution in America, Scandinavia, Europe and the Caucasus/Irano-Turanian, and (**C**) detailed occurrence of respective haplotype gene pools in Eurasia. Green stars indicate putative Botanical Gardens escapes. Shaded areas highlight the distribution of haplotypes (orange: H01-H08, green: H09-H17). Divergence time is taken from BEAST analysis (Fig. 2). Maps were generated in R using the mapdata package (A language and Environment for Statiscal Computing, R Core Team, R Foundation for Statiscal Computing, Vienna, Austria, 2017, https://www.R-project.org; mapdata: Extra Map Databases, R package version 2.2–6, https://CRAN.R-project.org/package=mapdata;), and figures were drawn in Inkscape v 0.91 (https://inkscape.org/).

All three genetic markers were successfully sequenced in 149 individuals and GenBank accession codes are provided (Suppl. Material Table 1). The original alignment which was including also some missing sequence information was 1809 bp in length (Suppl. Material Table 2) with 54 variable sites including the outgroup and 29 variable sites within *B*. *orientalis* only. The finally trimmed alignment was 1512 bp in length. In total 18 plastid haplotypes (H1-H17) were detected in *B*. *orientalis* and *B*. *erucago* as the outgroup. The haplotypes for individual accessions are indicated with Suppl. Material Table 1. A summary statistics for haplotype frequencies according to geographic regions is provided with Table 1. Parsimony network analysis shows a distinct European cluster of European haplotypes (H01-H05), which is separated from Irano-Turanian-Caucasian haplotypes (H09-H17) (Fig. 3A). Central haplotypes in the network, H07 and H08, are found in Turkey only, but haplotype H6, also found in the center of the network, not only occurs in northern Caucasus but is also found further westwards towards Central Europe. Respective divergence time estimates from BEAST analysis (Fig. 2) are indicated with the haplotype network (Fig. 3A). There is very strong biogeographic signal with the distribution of plastid haplotypes (Fig. 3B): in Eastern Turkey and adjacent regions including the Southern Caucasus plastid types H7-H17 were found exclusively. Exceptions are two recently introduced North American accessions, and two accessions from Western Europe close to Botanical Gardens (Wageningen, Netherlands, and Osnabrück, Germany). The frequency of haplotypes according to regions is indicated with Fig. 3A. Genetic diversity parameters sorted for different regions are shown with Table 2. For any geographic assignment of samples the Irano-Turanian-Caucasian region contributed most to the genetic diversity. As one would expect, regions colonized only very recently (North America or Scandinavia) show low genetic diversity. When we consider regional definitions following the division of Eurasia in areas of exotic (recently introduced but not invasive, Western Europe), invasive (introduced during the last 250 years and invasive) and native status of populations^16^, the first two regions carry low amounts of genetic variation compared to the proposed native range (Table 2).

**Table 1:** Distribution and frequency of plastid DNA haplotypes H1-H17 in the various regions.

	H1 (34)	H2 (51)	H3 (18)	H4 (2)	H5 (4)	H6 (9)	H7 (2)	H8 (1)	H9 (1)	H10 (2)	H11 (1)	H12 (6)	H13 (3)	H14 (3)	H15 (6)	H16 (5)	H17 (1)
Europe (61)	8	20	16	0	4	8	0	0	0	2	0	0	0	0	0	3	0
Scandinavia (41)	24	16	0	1	0	0	0	0	0	0	0	0	0	0	0	0	0
Caucasus-Irano-Turanian (25)	0	0	0	0	0	1	2	1	1	0	1	6	3	3	6	0	1
Asia (3)	0	2	0	1	0	0	0	0	0	0	0	0	0	0	0	0	0
North America (19)	2	13	2	0	0	0	0	0	0	0	0	0	0	0	0	2	0

**Table 2:** Summary statistics of plastid DNA based genetic diversity calculations grouped according to (A) geographic regions, (B) putative centers of origin, and (c) biological status.

	n	S	h	Hd	π	D
**(A)**						
North America	19	14	4	0.526	0.211	−0.7525
Scandinavia	41	2	3	0.517	0.065	2.0784*
Europe	52	6	5	0.755	0.139	1.4638
Caucasus-Irano-Turanian	25	24	10	**0.877**	**0.462**	0.3628
Asia	3	1	2	0.667	0.044	—
**(B)**						
Caucasus-Irano-Turanian*	24	24	9	**0.866**	**0.458**	0.1273
Europe	113	6	6	0.697	0.111	−1.0511
**(C)**						
Exotic	41	15	6	0.718	0.182	−0.6875
Invasive	67	6	6	0.677	0.104	0.6058
Native	29	25	11	**0.899**	**0.484**	0.3812

n = sample size, S = polymorphic sites, h = no. of haplotypes, Hd = haplotype diversity, π = nucleotide diversity, D = Tajima’s D (*significant at P < 0.05); *excludes haplotype H6 from northern Caucasus.

In summary, we can conclude that there are two geographically defined plastid gene pools, with the Irano-Turanian-Caucasian gene pool being more diverse and most likely ancestral to the European gene pool with highest haplotype and gene diversity (Table 2). The two gene pools are geographically and phylogenetically well-defined and mismatch-distribution analysis provides additional detailed temporal insights into the expansion of both of them. The Irano-Turanian-Caucasian gene pool expanded 0.93 Mya (Table 3), which is close to the deepest phylogenetic split detected among these haplotypes (1.22 Mya, Fig. 2). In contrast, the last obvious expansion of the European gene pool is calculated with 0.23 Mya and thus happened much later than split times among European haploytpes (0.3 Mya).

**Table 3:** Summary statistics from the mismatch distribution analyses of plastid DNA sequence variation.

	SSD (P)	HRag (P)	τ, mean (95% confidence interval)	Expansion time (Mya), mean (95% confidence interval)
Refuge areas:				
Caucasus-Irano-Turanian*	0.0526 (0.018)	0.0456 (0.197)	11.34 (4.65–16.44)	0.93 (0.38–1.35)
Europe	0.0691 (0.024)	0.1979 (0.045)	2.84 (1.04–5.44)	0.23 (0.08–0.45)

Using respective divergence time estimates within *Bunias* based on whole plastome sequence data (BEAST analysis, Fig. 2) the mean rate for the three loci under study has been re-calculated with μ = 2.004 × 10^−10^ mutations/site/year. *excludes haplotype H6 from northern Caucasus because of genetic intermediacy of this accession.

### Nuclear (AFLP) genetic assignment indicate strong genetic differentiation

The results from the initial population-based screening (401 individuals from 16 populations) are shown with Suppl. Material Fig. 1. The optimal numbers of genetic clusters (*K*) was estimated with 2 (Fig. S1D) and separates the same gene pools as demonstrated by plastid DNA analysis. This is also documented with congruent results from STRUCTURE and network analysis (Suppl. Material Fig. 1B). From this data set we also have the information for plastid haplotypes, and the results largely follows our expectations with two exceptional populations from Wageningen (Netherlands) and Osnabrück (Germany) carrying Irano-Turanian plastid haplotypes in a European nuclear genomic (AFLP) background. Both populations may represent garden escapes of unknown cultivation history. If we inspect an increased number of genetic groups (*K* = 9 shown in Suppl. Material Fig. 1D), populations largely remain as genetically uniform clusters demonstrating that within population genetic diversity is low and most variation is found between them. This screening provides the ratio to study herbarium vouchers with single individuals per population and indicates a minor bias towards under-sampled genetic variation on population level.

**Figure 4:**
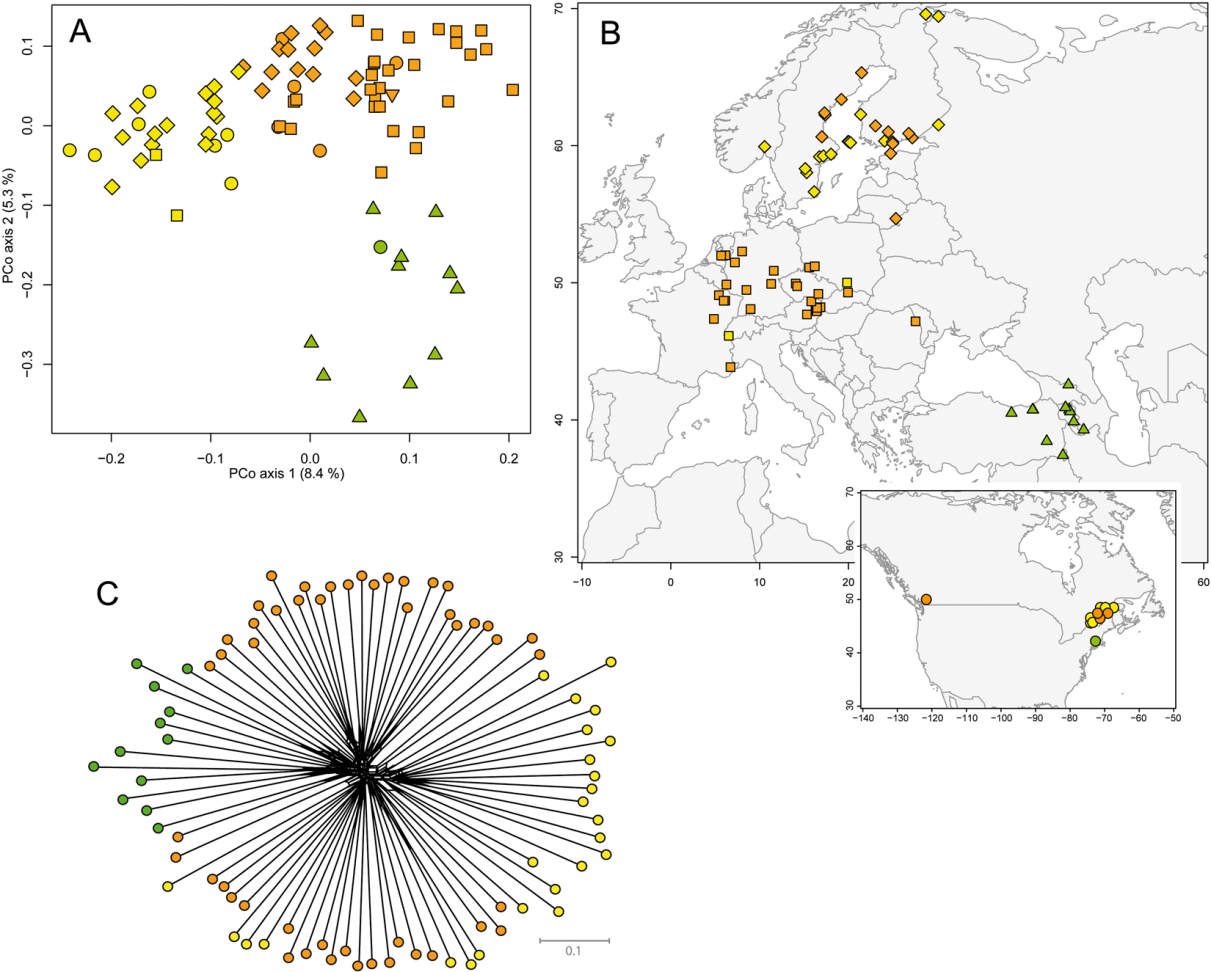
(**A**) PCoA analysis of AFLP data with the first two coordinates shown and the three gene pools indicated with color codes (yellow: Scandinavia, orange: Europe and Central Asia, green: Caucasus-Irano-Turanian) (Symbols code: circle: America, triangle: Caucaus-Irano-Turanian, box: Europe, diamond: Scandinavia, inverse triangle: Central Asia) (**B**) Geographic distribution of individually assigned AFLP genotypes. (**C**) SplitsTree graph of AFLP data (gene pools are indicated with colour code). Maps were generated in R using the mapdata package (A language and Environment for Statiscal Computing, R Core Team, R Foundation for Statiscal Computing, Vienna, Austria, 2017, https://www.R-project.org; mapdata: Extra Map Databases, R package version 2.2–6, https://CRAN.R-project.org/package=mapdata;), and figures were drawn in Inkscape v 0.91 (https://inkscape.org/).

Analysis of variance for the individual number of fragments in AFLP profiles generated from specimens collected prior to 2003 and samples collected thereafter showed a significant difference [F(1, 119) = 17.69, p < 0.001, Suppl. Material Fig. 2B]. The difference of variance for the number of fragments per individual for AFLP profiles generated on PCR plate 1 and PCR plate 2, however, was not significant [F(1, 119) = 1.0057, p = 0.318] and indicates no further experimental error. After quality control, removal of duplicate samples and filtering for low fragment number samples (excluding samples with a fragment number falling outside the range of µ + 2σ interval estimated from specimens collected 2003 onwards), 85 samples remained and the total number of sites scored was 294. The PCoA revealed 84 non-negative, non-zero eigenvalues. The first three eigenvalues were larger than the corresponding values obtained from the broken-stick model, accounting for 8.41%, 5.34% and 4.17% of total variation, respectively (cumulative: 17.92%) (Fig. 4, Suppl. Material Fig. 3C, Suppl. Material Table 2). Optimal numbers of genetic AFLP clusters increased from *K* = 2 to *K* = 3 compared to the population-based dataset with much fewer accessions (Suppl. Material Fig. 3A). The overall structure of the gene pools, however, remained very similar (Fig. 4, Suppl. Material Fig. 3B,C). The Caucasian-Irano-Turanian gene pool is separated from the European gene pool, the latter including accessions from Russia, Asia and mostly North America. The European cluster is divided into two subgroups, indicating differentiation of Central European and Scandinavia accessions (Fig. 4B). Aside accessions from Wageningen and Osnabrück we observed no additional accession with non-matching gene pools (AFLP versus plastid haplotype). Noteworthy, PCo 1 separates mostly European and Scandinavian accessions, and PCo 2 finally distinguishes between geographically (and plastid gene pool) defined Caucasian-Irano-Turanian accessions versus the rest. North American accessions cannot be differentiated as a whole and suggests multiple introductions from the European and the Caucasian-Irano-Turanian gene pool (Fig. 4B). However, a deep divergence between Scandinavian and Central European samples is not obvious (Fig. 4C). Genetic diversity statistics of AFLP genotypes according to regions (Suppl. Material Table 3a) indicate full congruence with plastid DNA based genetic parameters (Table 2) with the Caucasian/Irano-Turanian and Scandinavian regions showing highest and lowest genetic diversity values, respectively. In North America genetic diversity is higher than in Central Europe, which is fully explained by co-occurrence of genotypes from both gene pools. Similarly, AFLP fragment distribution among regions also indicates for example the highest number of unique fragments in the Caucasus-Irano-Turanian region (Suppl. Material Table 3b).

### Geographic and bioclimatic space analysis and European range expansion

Correlations among BIOCLIM variables 10 (mean temperature of the warmest quarter) and 11 (mean precipitation of the warmest quarter), 10 and 19 (mean precipitation of the coldest quarter), and 18 (mean temperature of the coldest quarter) and 19 were significant (p < 0.05), but not for variables 11 and 18. All correlations were weak (| r | < 0.5) and therefore all four variables were maintained at this stage. After forward model selection, only four model terms (spatial terms X^2^y, y, x and y^2^) were retained. Thus, the final model formula was ~X^2^y + y + x + y^2^ + condition (gene pool: Caucasian-Irano-Turanian versus European). Variance inflation factors then ranged in 1.259 and 2.575^31^. In the final model the proportion of conditional variance (gene pool) was 22.83% of total variance, proportion of constrained variance (spatial terms) was 27.03% of total variance, and residual variance (not accounted for by the model) was 50.13%. The observed value of F for the whole model was significant in 1000 permutations, F_(4, 79)_ = 10.649, p_(F’> F)_ ≤ 0.001 (Table 4). RDA axes 1 to 3 explained 62%, 25% and 13% of constrained variation respectively; the pseudo F-ratio was significant for every axis in 1000 random permutations (Suppl. Material Fig. 4).

**Table 4:** Proportion of total genetic variance explained by partial redundancy analysis (RDA); the variance component due to phylogenetic relatedness among individuals has been partialled out as a conditional term.

model term	df	% of total variation	variance	P(F’ > F)
X^2^Y	1	5.4	0.0019163	≤ 0.001
Y	1	14.0	0.0049961	≤ 0.001
X	1	5.2	0.0018415	≤ 0.001
Y^2^	1	2.5	0.0009008	≤ 0.011
Gene pool (condition)	1	22.8	0.008154	
Residual	79	50.1	0.017905	
total	84	100.0	0.035714	

In summary, RDA was used to search for parameters that account for genetic structure in the AFLP dataset: (a) climatic parameters, (b) spatial origin, (c) ancestral gene pools (conditional term). Our results did not show a significant signal that bioclimatic variables can sufficiently describe patterns of genetic structure of gene pools (considering spatial independency), which is in particular true for the Central European region; however, a clear signal of genetic isolation by distance can be found, e.g., when comparing Central European and Scandinavian accessions (Suppl. Material Fig. 4).

## Discussion

This study shed light on the unknown evolutionary history of the biologically very interesting enigmatic *B*. *orientalis*. Why that? The Brassicaceae with its nearly 4000 species are grouped into few major evolutionary lineages and further into 52 monophyletic tribes. Most of these tribes originated (stem group age) approximately 20 Mya with differing ages of crown group radiation often leading to species-rich and more rarely species-poor clades^20^. The only two species from tribe Buniadeae, *Bunias erucago* and *B*. *orientalis*, have a primarily (Eastern) Mediterranean-Asian distribution. There is no explanation for why this lineage did not radiate, and maybe this is related to the observation that genome size is very large compared to the average genome size in Brassiaceae thereby hindering genetic differentiation. Chromosome number is 2n = 14 and genome size is of about 2 GBp, which is 15X times larger than *Arabidopsis thaliana* and 5X larger than assumed ancestral crucifer genome size)^32^. In contrast, *B*. *orientalis* is one of the various perennial successful world-wide colonizers among Brassicaceae, and *B*. *orientalis* is among the very few Brassicaceae species categorized as invasive not only in Europe but also the United States. Therefore, *B*. *orientalis* is an excellent system to study sudden and explosive range expansion under severe environmental change (last 200 years). However, this requires elucidating its evolutionary history in order to formulate and test hypothetical causes of recent range expansions and respective adaptive processes, and to infer phylogenetic-evolutionary constraints explaining present-days processes^33,34^. Herein, we show that the tribe Buniadeae originated approximately 12 Mya, and the split between the only members of the tribe, *B*. *orientalis* and *B*. *erucago*, dates back 5 Mya, resulting in 7 My of net diversification rate of zero. The species split correlates well with the end of the Messian desiccation crisis (7.3 to 5.3 Mya) in the Mediterranean at the Miocene-Pliocene climatic transition and further confirms the observation that diversification events in Brassicaceae are often correlated with major environmental transitions^20,21^.

Previous studies provided a hypothesis about the extent of the native range of *B*. *orientalis* ^16^ reaching from Eastern Europe to Central Asia including the Caucasus, the Irano-Turanian region and Central Asia (Fig. 1). Our results indicate that the spatial distribution of *B*. *orientalis* gene pools is more complex. The onset of *B*. *orientalis* diversification is calculated with 1.21 Mya and separates an old Irano-Turanian-Caucasian gene pool from a European gene pool. This Irano-Turanian-Caucasian gene pool did not show significant range shifts and a mismatch analysis indicates that its expansion followed during the Early-Mid-Pleistocene *c*. 0.93 Mya. During the “middle Pleistocene transition” (1.2–0.8 Mya) global cold period climates intensified, which coincides with the 100 ka Milankovitch cycles causing severe cold periods allowing the development of continental-scale ice sheets^35^. There are only very few estimations of divergence and expansion times for plants from this region. In case of the genus *Aubrieta* Adans (Brassicaceae) which has a distribution and diversity center in Anatolia and the Irano-Turanian region, the onset of expansion-radiation of the entire genus was estimated to be 1.17 Mya. The P-S-M group (taxa from Eastern Turkey and distributed further towards Iran and Iraq) started to expand 1.11 Mya^36^. This phylogeographic expansion pattern was correlated with niche shifts towards higher temperature and larger temperature ranges^36^. A similar pattern has been described for *Arabis alpina* ^37^, which grows sympatrically at many places with different *Aubrieta* species. The diversification of Anatolian genetic diversity of the *A*. *alpina* complex was calculated at 0.7 Mya^37^.

The “native” range of the European gene pool (Figs 3,4) largely correlates with the Eastern European region hypothesized earlier^16^ and is redrawn here (Fig. 1). However, few details are notably: (1) in the Northern Caucasus is the only accession from the Irano-Turanian gene pool carrying plastid haplotype H6, which mediates between both plastid gene pools, (2) Asian accessions (Russian Altai, Uzbekistan) also belong to the European gene pool.

The Caucasus is of particular interest, because it is one of the global biodiversity hotspots^38^ and harbors an extraordinary biological diversity, including plant species that are meanhwile considered invasive elsewhere (e.g., *Heracleum mantegazzianun*, *Veronica persica*, *Bunias orientalis*, *Phedimus spurius*). The region connects Europe and Asia both biologically and culturally. Caucasian ecosystems encompass moist forests at the coasts of the Black and Caspian Seas (Arcto-Tertiary forests), open and xerophytic woodlands, mountain habitat types including meadows as well as steppes that become more dominant towards the East. It is also important to note that many aspects of the Caucasian biodiversity patterns can only be understood in a wider geographical context because the Caucasus is a “melting pot” of elements of different floristic regions with different histories and origins. The Caucasus is particularly interesting because it lies at the border of the Euro-Siberian and Irano-Turanian floristic regions and harbors elements from both floras. Understanding the evolutionary history and origin of the Caucasian flora will therefore also allow for a better understanding of floristic connections in Eurasian mountains, which have been hypothesized based on floristic studies but not yet shown in biogeographic analyses^39^. Our data demonstrate that *Bunias* originated south of the Caucasus, but it is likely that *B*. *orientalis* is an indigenous floristic element of open grassland in mountainous and highland-(sub)alpine regions of the Southern Caucasus. This idea has been mentioned earlier^17–19^, but others proposed a much wider original distribution range including South and Central Russia, West Siberia and southeastern Europe up to the southern borders of contemporary Slovakia and east Hungary^18,40,41^. However, mismatch distribution analysis of the European gene pool demonstrates an expansion time of about 230 kya, which is predating the maximum ice-sheet extension during the Saale-Riss glaciation approximately 150 kya, which severely affected also the Northern Caucasus. The northernmost population analyzed herein from the Caucasus region (North Georgia) carries a rare European haplotype (H6). This might indicate that the mountain ranges in Northern Georgia served as strong barrier after maximum glaciation 150 kya, which gave rise to a secondary distribution area north of the Caucasus that reached south and central Russia, west Siberia and southeast Europe. However, with our data we cannot define precisely a geographic region of this secondary center of distribution and genetic diversity. There is very little macrofossil evidence of *Bunias* to test for spatial distribution pattern independently, but there are plant remains of *B*. *orientalis* from Northwest Altai from the 4^th^ and 3^rd^ century BC, which were also confirmed by DNA sequence data^42^. Published DNA sequence data from this material are from ITS (internal transcribed spacers of nuclear ribosomal DNA), but unfortunately we found no variation among all of our accessions (data not shown) and cannot assign this old material to any gene pool. This Scythian burials are interpreted in a way that Altai people at around the foothills used this plant in common and ritual fields and it must be assumed that the species has been widely distributed in Siberia and Altai and went extinct afterwards, long before the 19^th^ and 20^th^ century and the start of present-day expansion history.

Our bioclimatic analysis supports these ideas, because if we remove the biogeographic component (explains 40% of genetic differentiation) from PCoA, bioclimatic variables explain only 2.6% of the structure of genetic variation, and RDA indicates that a large proportion of structure in genetic variation is best explained by isolation-by-distance. If we consider that the phylogenetic diversity of a species mainly follows a biogeographic signal, then it is noteworthy that bioclimatic parameters do not explain Europe-wide genetic differentiation patterns (e.g., Central Europe *versus* Scandinavia) nor differentiate among the gene pools and it remains open what the causal factors are that drive populations in Central Europe to become invasive.

This study also highlights the ongoing value of herbarium vouchers for two reasons: (1) hardly accessible areas have been sampled, for which herbaria very often offer the unique opportunity to collect reliably material with detailed collection data^43^; (2) potentially important material from the recent past, often highlighting introduction history, is available^44^. There is, of course, a quality effect of herbarium vouchers, but this does not exclude “old” samples a priori as we have shown in this study^45^. We have successfully analyzed samples collected from within the last 81 years, which makes herbaria a valuable treasure trove for quantification of past (historical) genetic diversity. In our data set this is best exemplified by North American samples. *Bunias orientalis* has been introduced into Canada around 1944^22,46^ and into the US in the 1950s^23,24,46^. Our samples span the Northern US and Southern Canada and indicate a diverse genetic set-up. We identified a voucher from the US (Ann Arbor, Michigan), which has been collected in 1936 and notes “Abundant and well established in low meadows”. This indicates that published documentations miss 20 years of potential introduction history and that herbarium vouchers are important long-term archives for such kind of knowledge with its physical documentations. Accessions from the northwestern US carry plastid and AFLP genotypes from the Caucasian-Irano-Turanian gene pool, whereas Canadian accessions exclusively show European plastid haplotypes H1, H2 and H3 and a respective genomic background. These findings correspond well with independent, multiple introductions into the North American continent, and it remains important for the next future to follow potential amalgamation of these two gene pools with additional adaptive potential for further and increased invasiveness (*Reynoutria* in Europe as an example^47^).

The rapid range expansion in Western Europe and Central Europe is not accompanied with a decrease of genetic diversity in this regions. Considering that eventually *B*. *orientalis* expanded into Central Europe from the adjacent regions of the Caucasus not earlier than the 18^th^ century^40,48^ and having in mind that the species has already successfully invaded into most parts of Central Europe since the second half of the nineteenth century, genetic pattern suggest multiple and also long distance dispersals all over the European distribution range thereby blurring any regional spatial pattern of genetic diversity. The example from Asian Altai demonstrate that we cannot exclude that *B*. *orientalis* entered Eastern-Central Europe earlier than the 18^th^ century, but it is obvious that human corridors massively promoted the species’ spreading (grassland along rivers, roads, railways, fields)^18,27,41^.

This work is not focusing on the recent invasion history of the *B*. *orientalis*, but we provide a comprehensive evolutionary framework any future study can rely on. The species does not show a pattern of leading edge colonization, but a mixture of continuous long and mid-range dispersal all over Central Europe. Since we did not observe a climatic signature explaining neither distribution nor genetic variation, but also having climatic gradients in the species’ distribution range (e.g., from the Atlantic coast to Pannonical drylands), we might have to assume a greater potential to adapt locally. This fits well with missing evidence for significant shifts in ecological traits comparing plants of non-invasive (exotic) and invasive populations/regions^25^ (see also Fig. 1). However, invasion success may be facilitated by high chemical diversity as insect herbivory defense mechanism, which is directly correlated with genetic diversity^49^ either as a result of multiple introduction or amalgamation of local gene pools. Therefore, spreading of gene pools such as from the Caucasian-Irano-Turanian region should be seriously monitored and documented and may serve also as a great opportunity to learn more about fundamental evolutionary processes during rapid range expansion.

## Methods

### Plant material and DNA isolation

A first comprehensive screening of genetic variation on population level was done with 16 populations (401 individuals in total) of *Bunias orientalis* selected from the entire distribution range (Suppl. Material Table 1). Results from this pre-screening provided the rationale to collect densely all over the distribution area of *B*. *orientalis* focusing on herbarium vouchers. Finally, 114 additional accessions from the entire distribution range were obtained with precise geographic sampling data (Suppl. Material Table 1, Fig. 1). The genus *Bunias* comprises two species only, and *B*. *erucago* was used as outgroup in plastid haplotype network analysis^50,51^. Total genomic DNA was extracted from herbarium vouchers, silica dried or fresh leaf material either following the CTAB protocol by^52^ or using the Invisorb Spin Plant Mini kit (STRATEC Biomedical AG, Birkenfeld, Germany).

### Plastid genome analysis

In order to provide a temporal framework of the evolutionary history of the genus *Bunias* and to obtain values for molecular mutation rates for further genetic data analysis (e.g., mismatch distribution analysis), six *Bunias* samples (*B*. *erucago* plus five *B*. *orientalis* accessions: BO26, B0118, T417, OS09, RO12) were selected for sequencing and annotating their entire plastid genomes (corresponding to haplotypes H6, H6, H9, H16 and H2, respectively; see below). Plastome sequence data from *Hesperis matronalis*, *Clausia aprica* (also newly generated herein and from the same evolutionary lineage as *Bunias*) were selected for further analysis, because this evolutionary lineage was under-represented in prior studies^20^. The newly sequenced genome data were incorporated into a Brassicaceae family and angiosperm wide plastome data set^20^ in order to obtain stem and crown group ages. The lack of reliable fossil evidence for time calibration in Brassicaceae makes it necessary to choose outgroups outside the family. Details of library preparation, assembly and annotation have been documented earlier^20^. Further details on plastome assembly, alignment preparation using MAFFT v.7.017^53^,^54^, phylogentic analysis and divergence time analysis using BEAST^55^ are provided with Supplementary material text.

### Plastid haplotype analysis

Based on plastid genome sequence information, we selected three informative regions with a sufficient number of SNPs (single nucleotide polymorphisms) to reconstruct haplotype networks based on the entire set of accessions and individuals. (1) *trn*L^UAA^-intron, (2) *trn*L^UAA^-*trn*F^GAA^ intergenic spacer, and (3) *trn*G^UCC^ intron^56,57^. Details on primer sequences and PCR conditions used are provided with the Supplement. Parsimony Network analysis of concatenated alignemnts of these three plastid sequences, defined as discrete haplotypes, was performed using the programs TCS v.1.21^58^ and SplitsTree4 v. 4.14^59^. Length variation in polyA, T, or G regions were not considered and not coded as characters.

In order to test a demographic expansion scenario, mismatch-distribution analysis^60^ of plastid sequence data was performed using a sudden (stepwise) expansion model^61^ of the main regions. Goodness-of-fit was tested using the sum of squared deviations (SSD) between observed and expected mismatch distributions and Harpending’s raggedness index (HRag)^62^. For groups with expanding populations, the expansion parameter (τ) was converted to an estimate of time (T, in number of generations) since the start of expansion began using T = τ/2 u^60,63^. The neutral mutation rate for the concatenated sequence (haplotype) per generation of u is calculated as u = μkg, where μ is the substitution rate, k is the average sequence length of the DNA region under study, and g is the generation time in years. Generation time g was set to two years. Total alignment length was 1512 base pairs. The substitution rate was set to 2.004 × 10^−10^ mutations/site/year using the crown group age of the genus (4.95 Mya; results from BEAST analysis, see above) and referring to the three-marker-set. A parametric bootstrap approach^61^ with 1000 replicates was used to assess the goodness-of-fit of the observed mismatch distribution to the sudden expansion model, to test the significance of HRag, and to obtain 95% confidence intervals (CIs) around τ. Tajima’s D^64^ tests of selective neutrality were conducted. All the above analyses were carried out with Arlequin 3.5.2.2.^65^ and DNAsp^66^.

### Genetic diversity and nuclear AFLP analysis

AFLP profiles were generated for the population dataset with 401 individuals from 16 populations (Supplementary Material Table 1) using three combinations of selective primers (Supplementary Material text). To assess technical genotyping error, we included replicate samples from duplicated DNA extractions in our setup. In total, we generated 100 replicate genotypes for 55 individuals. Samples were assigned randomly to an experimental block (96 well reaction plate). Rate of genotyping error was determined over all pairs of replicates^67^. The large-scale analysis included 134 accessions of which 25 individuals representing 16 populations from the population dataset were serving as internal controls to compare results among experiments (134 genotypes, 404 loci after first screening using GeneMarker 1.95 (SoftGenetics LLC, State College, USA) (Supplementary Material Table 1, Fig. 1). Details of the AFLP protocol^68,69^ are given with Supplementary material text.

The calculated error rate yielded high reliability and reproducibility of the fingerprints (1.1% of the population data set, and of 2.2% for the large dataset). Since herbarium vouchers often provide varying qualities of DNAs that may affect AFLP banding patterns, the large-scale herbarium voucher dataset was analyzed further to improve data quality. We tested for homogeneity of variance (HOV) of the number of AFLP fragments generated for every individual specimen using the Brown-Forsythe test^70^ implemented in the R package *car* ^71^. Alpha was adjusted for multiple comparisons using Bonferroni’s correction^72^. For final ordination analysis, we excluded samples with a number of fragments falling outside the range of the µ + 2σ interval estimated from specimens collected after year 2002. Associations of all pairs of samples were expressed by a dissimilarity coefficient with Euclidean properties^72,73^). Principle Coordinate Analysis (PCoA)^74^ and eigendecomposition of the resulting association matrix was performed with the eigen() function in R^75^.

Genetic assignment of accessions was inferred under the admixture model implemented in STRUCTURE 2.3.4^76^. using the correlated allele frequencies and the recessive alleles options^77^. Burnin period comprised 150,000 MCMC steps and data collection was carried out over another 250,000 steps. Ten replicate simulations were run for each value of *K* (the number of ancestral clusters assumed by STRUCTURE) ranging from 1 to 10. Individual ancestry was averaged across all replicate simulations for each value of *K* using CLUMPP Version 1.1.2^78^. A formal determination of the optimal value of *K* was carried out using Evanno’s Mean Delta *K* ^79^ and Symmetric Similarity Coefficients ^80,81^

Analysis of Molecular Variance (AMOVA) and estimation of molecular diversity indices were performed with Arlequin v. 3.5^65^. SplitsTree graphs were generated with SplitsTree4 v. 4.14^59^.

### Geographic and bioclimatic differentiation: Redundancy analysis (RDA)

We performed (partial) redundancy analysis (RDA)^82,83^ on a data matrix comprising significant eigenvectors obtained by PCoA of the association matrix of genetic dissimilarities (AFLP dataset). The set of constraining variables comprised (in this order) nine 3^rd^ degree orthogonal polynomials of geographic coordinates^84^ and BIOCLIM variables 10, 11, 18 and 19 at a spatial resolution of 10 arc minutes (mean temperature and precipitation of the warmest and coldest quarter respectively^85^. Environmental variables were centered on the mean and scaled to standard deviation (Z standardization). Latitude and longitude were Z standardized prior to calculating the polynomials. A binary factor coding whether a specimen’s plastid DNA type belonged to either the Caucasus-Irano-Turanian or the European gene pool was used as a conditional term. Since the corresponding split in the plastid gene pools clearly pre-dated the time frame of the historic range expansion of European *B*. *orientalis*, we treated phylogenetic relatedness among individuals as a (undesired) covariate.

All statistical analyses were performed in R^75^ using the corresponding functions from the R package *vegan* ^86^ to perform RDA, forward model selection, permutation based significance tests and variance inflation factors. We performed forward model selection on a full model comprising all explanatory variables (9 spatial + 4 climate + 1 condition) and all pairwise interactions terms between all pairs of spatial and environmental terms (N = 4 × 9 = 36). A model term was included in the final model, if the proportion of pseudo F-ratios F’ that were larger than the observed pseudo F did not exceed α = 0.01 in 1000 random permutations^87^.

### Data Availability

The datasets generated and analysed during the current study are available from GeneBank and from the corresponding author on reasonable request.

## Electronic supplementary material

Supplementary Information
