# Supplementary material for: Early-Mid Pleistocene genetic differentiation and range expansions as exemplified by invasive Eurasian Bunias orientalis (Brassicaceae) indicates the Caucasus as key region
Source: Sci Rep. 2017 Dec 1;7:16764. doi: 10.1038/s41598-017-17085-8 (PMC5711908; doi:10.1038/s41598-017-17085-8)
Supplement: Supplementary file 1 — Supplementary Information [file 41598_2017_17085_MOESM1_ESM.pdf]

Supplementary material to:

Early-Mid Pleistocene genetic differentation and range expansions as exemplified by invasive Eurasian *Bunias orientalis* (Brassicaceae) indicates the Caucasus as key region.

Marcus A. Koch[1, *], Florian Michling[1,+], Andrea Walther[1,+], Xiao-Chen Huang[1,+], Lisa Tewes², and Caroline Müller²

[1]Heidelberg University, Centre for Organismal Studies, Heidelberg, 69120, Germany
[2]Bielefeld University, Chemical Ecology, Bielefeld, 33615, Germany
*corresponding author: marcus.koch@cos.uni-heidelberg.de
[+]these authors contributed equally to the work

Content:

Expanded Material & Method section (incl. literature) **p.1**

Supplementary Material Figure 1: Population-based AFLP screening **p. 8**

Supplementary Material Figure 2: AFLP screening results from total dataset **p. 9**

Supplementary Material Figure 3: AFLP results, K-estimation in genetic structure **p. 10**

Supplementary Material Figure 4: Results from RDA analysis **p. 11**

Supplementary Material Table 1: Accession details **p. 12**

Supplementary Material Table 2: PCoA details for AFLP analysis **p. 15**

Supplementary Material Table 3: AFLP genetic data summary statistics **p. 16**

Details of DNA extraction methods

Dried leaf material was taken from herbarium vouchers. Material was homogenized either by grinding with a pistil on liquid nitrogen, or using a Precellys®24 homogenizer (Bertin Technologies, Montigny-le-Brettonneux, France) at 5,000 rounds per minute for two times 10 seconds with five 2.5 mm glass beads. The Invisorb Spin Plant Mini kit was used following the manufacturer's instructions, including the optional step of RNA digestion. The following modifications were applied to the CTAB protocol: DNA pellets were washed twice with 70% ethanol and then dissolved in 100 µl TE-buffer (10 mM Tris-HCl, 1 mM EDTA, pH 7.5) supplemented with 2 units of RNase A, RNA digestion was performed at 37 °C for 1 h. Quality (high molecular weight) and quantity of DNA was checked prior subsequent analytical steps. DNA quality and fragment length was checked on 1% agarose gels, and concentration was assessed via fluorescence spectroscopy using a high-sensitivity, double-stranded DNA specific dye with the Qubit® dsDNA HS Assay (Thermo Fisher Scientific, Waltham, Massachusetts, USA).

Plastid genome assembly and detailed description of data handling and divergence time analysis

Complete chloroplast sequences were aligned using MAFFT v7.017 (Katoh et al., 2002; Katoh, 2005) implemented in Geneious v7.1.7 (Biomatters Ltd., Auckland, New Zealand). The FFT-NS-ix1000 algorithm was used, with the 200 PAM/k = 2 scoring matrix, a gap open penalty of 1.53 and an offset value of 0.123. Alignments were constructed for every order separately, or, if not possible because of major rearrangements in the order of genes, for smaller datasets. Coding sequences (CDS) were then extracted from the alignments according to published annotations, and introns were excluded. Finally, the gene alignments of all orders were combined, and genes with no missing data were selected; pseudogenes were excluded. This resulted in a set of 73 genes, including 51 protein-coding genes, 19 tRNAs, and three rRNAs. All genes were realigned (settings as above) and checked manually, and for protein-coding genes start and stop codons were excluded from further analyses because of their high potential for homoplasy. Indels were excluded from the alignments using Gblocks v0.91b (Castresana, 2000). The minimum length of a block was set to 2 bp, and because of the high sequence divergence in the dataset non-conserved blocks were also saved. To account for rate heterogeneity among genes, the dataset was partitioned into subsets of genes evolving at a similar rate under the same substitution model using PartitionFinder v1.1.1 (Lanfear et al., 2012, 2014). Branch lengths were

allowed to be unlinked, and only models implemented in BEAST were tested, with BIC used for model selection in a greedy search.

Divergence time estimation followed our earlier strategy (Hohmann et al., 2015) and was conducted in BEAST v1.7.5 (Drummond et al., 2012) using independent site and clock models, but a combined partition tree for the three partitions (GTR+I+Γ). *Vitis* was defined as outgroup. The ML tree was used as a starting tree after it was made ultrametric with node ages to fit constraints using the R package APE version 3.1-4 (Paradis et al., 2004). We tested the hypothesis that a molecular clock could be fitted to our data by applying a Chi-square test to lnL values of distance trees with Enforced Clock (EC) and Without Clock (WC). Our plastome dataset significantly rejected a strict molecular clock hypothesis (df=84, 2lnL(EC-WC)=42681.369, P=0). Therefore, an uncorrelated lognormal relaxed clock was applied . with estimated rates to account for rate variation among branches (Drummond et al., 2006). We chose four fossil constraints that were also used in the most recent and comprehensive angiosperm-wide temporal analyses and were in accordance with Magallón et al. (2015). Following Njugana et al. (2013), the minimum age for the *Prunus/Malus* split was set to 48.4 mya (Benedict et al., 2011), and the *Castanea/Cucumis* split to 84 mya (Sims et al., 1999; Moore et al., 2010). Following Bell et al. (2010), the minimum age for *Mangifera*/*Citrus* was set to 65 mya (Knobloch and Mai, 1986) and for *Oenothera*/*Eucalyptus* to 88.2 mya (Takahashi et al., 1999). A uniform distribution was used for all four fossil constraints with a maximum age of 125 mya. We also tested whether exponential or log normal distributions of fossil calibrations resulted in different age estimates by running the same analyses but with 100,000,000 generations. However, no substantial differences were found, so we chose uniform distributions, which use the least assumptions and assume the same model used in the most recent contributions (Beilstein et al., 2010; Hohmann et al., 2015). The root was calibrated with a uniform distribution between 92 and 125 Ma (minimum age of 125 mya to crown eudicots; Magallón et al., 2015). We ran two independent MCMC runs with 500,000,000 generations each and sampling parameters every 50,000 generations. The "Birth-death with incomplete sampling" model was used according to the characteristic of the dataset. This follow a previous study focusing on elaborating a timeline of Brassicaceae evolutionary history with a similar angiosperm-wide dataset (Hohmann et al., 2015). LogCombiner v1.7.5 (Drummond et al., 2012) was used to combine trees from the two runs and the first 50,000,000 generations of each run were discarded as burn-in. The resulting 18,000 trees were combined to a maximum clade credibility tree

in TreeAnnotator v1.7.5 (Drummond et al., 2012) and visualized in FigTree v1.4.1 (Drummond et al., 2012).

Primer details and PCR conditions for plastid haplotype detection

Based on plastid genome information we selected three informative regions with a sufficient number of SNPs (single nucleotide polymorphisms) to reconstruct haplotype networks based on the entire set of accessions and individuals. (1) *trn*L^{UAA} intron using primer pair 5´-CGA AAT CGG TAG ACG CTA CG-3´and 5´-GGG GAT AGA GGG ACT TGA AC-3´(Shaw et al. 2005, Taberlet et al. 1991), (2) *trn*L^{UAA}-*trn*F^{GAA} intergenic spacer using primer pair 5´-GGT TCA AGT CCC TCT ATC CC-3´ (Shaw 2005, Taberlet et al. 1991) and a reverse primer 5´-TCC TCT GCC AAG AAC CAG ATT TG-3´ matching to the *trn*F^{GAA} 3' CDS (Tewes et al., 2017), and (3) *trn*G^{UCC} intron using primer pair *trn*G^{UCC}F (GTA GCG GGT ATA GTT TAG TGG T) and *trn*G^{UCC}R (GGG TAG CGG GAA TCG AAC) (Tewes et al., 2017). Forward primers carried the M13uni (-21) extension (TGT AAA ACG ACG GCC AGT) and reverse primers the M13 rev (-29) extension CAG GAA ACA GCT ATG ACC, respectively, for subsequent custom sequencing. PCR reactions were performed with 1 µL template DNA in a final volume of 25 µL. Reagents were at final concentrations of 0.128 µM (each primer), 1.7 mM ($MgCl_2$), 0.388 µM (dNTP, species at equal proportions) and 0.6 U of MangoTaq DNA Polymerase in 1x concentrated MangoTaq Colorless Reaction Buffer (both Bioline, Luckenwalde, Germany). Thermocycling conditions basically followed Shaw et al. (2005) with few minor modifications: *trn*LF intergenic spacer: 95°C, 3 min; 30X (95°C, 30s; 50°C, 30 sec; 72°C, 1 min); 72°C, 10 min; *trn*L-intron: 95°C, 2 min; 30X (95°C, 30s; 50°C, 30 sec; 72°C, 1 min); 68°C, 10 min; *trn*G intron: 80°C, 5 min; 35X (95°C, 1 min; 50°C, 1 min; 65°C, 5 min); 65°C, 5 min. PCR products were purified using a NucleoFast 96 PCR plate ultrafiltration kit (Macherey-Nagel). DNA custom sequencing was performed by GATC (Konstanz, Germany) and MWG Eurofins, Ebersberg, Germany).

Details on AFLP protocols

Digestion of diluted genomic DNA and ligation of dsDNA adaptors was performed simultaneously by endonucleases *Eco*RI HF and *Mse*I and T4 DNA Ligase (New England Biolabs GmbH, Frankfurt am Main, Germany). PCR reactions were carried out with AmpliTaq DNA Polymerase in AmpliTag buffer II for PCR step 1 and with

AmpliTaq Gold and AmpliTaq Gold Buffer (all Life Technologies, Darmstadt, Germany) for PCR step 2 (selective PCR). Oligonucleotides and fluorescent-labelled oligonucleotides (with fluorophores FAM, TAMRA and TET) were obtained from biomers.net GmbH Ulm, Germany. During an initial screening for variability, a set of three combinations of selective primers was chosen for the study: (A) EcoRI+AAC(FAM)/MseI+CAG, (B) EcoRI+AAC(TAMRA)/MseI+CGG and (C) EcoRI+AGC (TET)/MseI+CAG). A detailed protocol of the laboratory routines including reagent concentrations and thermocycling conditions is given in Tewes et al. (2017). Prior to fragment detection, amplicons were poolplexed and purified by ultrafiltration (NucleoFast 96 PCR plate ultrafiltration kit, Macherery-Nagel). Fragment detection was performed on an Amersham MegaBace 1000 DNA Analysis System (GE Healthcare GmbH, Solingen, Germany). Size Calling and Manual Genotype Calling were performed with GeneMarker 1.95 (SoftGenetics LLC, State College, USA). Scored data were exported as a binary data table for further analysis (401 genotypes, 201 loci).

The large-scale analysis included 134 accessions of which 25 individuals/16 popuations from the populational dataset were serving as internal controls to compare results among experiments (134 genotypes, 404 loci after first screening using GeneMarker 1.95 (SoftGenetics LLC, State College, USA) (Supplementary Material Table 1, Fig. 1). For further optimization of fragment´s flourescent signal detection using custom services (GATC, Konstanz, Germany) TAMRA and TET were replaced by HEX and ATTO550, respectively, and six additional primer combinbations were selected: EcoRI+AAC(FAM)/MseI+CAG (original A), EcoRI+AAC(HEX)/MseI+CGG (original B), EcoRI+AGC (ATTO550)/MseI+CAG (original C), EcoRI+AAC(FAM)/MseI+CGC, EcoRI+AAC(HEX)/MseI+CGT, EcoRI+AGC (ATTO550)/MseI+CGA), EcoRI+ACG(FAM)/MseI+CAG, EcoRI+AAC(HEX)/MseI+CGA, EcoRI+AGC (ATTO550)/MseI+CGC.

Beilstein Beilstein, M.A., Nagalingum, N.S., Clements, M.D., Manchester, S.R., & Mathews, S. Dated molecular phylogenies indicate a Miocene origin for *Arabidopsis thaliana*. *Proc. Natl. Acad. Sci. USA* **107,** 18724–18728 (2000).

Benedict, J. C., DeVore, M. L., & Pigg, K. B. *Prunus* and *Oemleria* (Rosaceae) flowers from the late early Eocene Republic flora of Northeastern Washington State, U.S.A. *Int. J. Pl. Sci.* **172,** 948–958 (2011).

Castresana, J. Selection of conserved blocks from multiple alignments for their use in phylogenetic analysis. *Molec. Biol. Evol*. **17,** 540–552 (2000).

Drummond, A. J., Ho, S. Y. W., Phillips, M. J. & Rambaut, A. Relaxed phylogenetics and dating with confidence. *PLoS Biol*. **4,** e88 (2006).

Drummond, A. J., Suchard, M. A., Xie, D. & Rambaut, A. Bayesian phylogenetics with BEAUti and the BEAST 1.7. *Molec. Biol. Evol.* **29,** 1969–1973 (2012).

Hohmann, N., Wolf, E. M., Lysak, M. & Koch, M A. A time-calibrated road map of Brassicaceae species radiation and evolutionary history. *Pl. Cell* **27,** 2770–2784 (2015).

Katoh, K., Misawa, K., Kuma, K. & Miyata, T. 2002. MAFFT: a novel method for rapid multiple sequence alignment based on fast Fourier transform. *Nucleic Acids Res*. **30,** 3059–3066 (2002).

Katoh, K., Kuma, K., Toh, H. & Miyata, T. MAFFT version 5: improvement in accuracy of multiple sequence alignment. *Nucleic Acids Res*. **33,** 511–518 (2005).

Knobloch, E. D. & Mai, D. H. 1986. Monograph of the fruits and seeds in the Cretaceous of Central Europe. *Rozpravy Ústreõdního Ústavu Geologickéh* **47,** 1–219 (1986).

Lanfear, R., Calcott, B., Ho, S. Y. & and Guindon, S. PartitionFinder: combined selection of partitioning schemes and substitution models for phylogenetic analyses. *Molec. Biol. Evol*. **29,** 1695–1701 (2012).

Lanfear, R., Calcott, B., Kainer, D., Mayer, C. & Stamatakis, A. Selecting optimal partitioning schemes for phylogenomic datasets. *BMC Evol. Biol.* **14,** e82 (2014).

Magallón, S., Gómez-Acevedo, S., Sánchez-Reyes, L. L. & Hernández-Hernández, T. 2015. A metacalibrated time-tree documents the early rise of flowering plant phylogenetic diversity. *New Phytol.* **207,** 437–453 (2015).

Moore, M. J., Soltis, P. S., Bell, C. D., Burleigh, J. G. & Soltis, D. E. Phylogenetic analysis of 83 plastid genes further resolves the early diversification of eudicots. *Proc. Natl. Acad. Sci. USA* **107,** 4623–4628 (2010).

Njuguna, W., Liston, A., Cronn, R., Ashman, T. L. & Bassil, N. Insights into phylogeny, sex function and age of *Fragaria* based on whole chloroplast genome sequencing. *Mol. Phylogenet. Evol*. **66,** 17–29 (2013).

Shaw, J. et al. The tortoise and the hare II: relative utility of 21 noncoding chloroplast DNA sequences for phylogenetic analysis. *Am. J. Bot*. **92,** 142–166 (2005).

Sims, H. J., Herendeen, P. S., Lupia, R., Christopher, R.A. & Crane, P. R. Fossil flowers with Normapolles pollen from the Upper Cretaceous of southeastern North America. *Rev. Palaeobot. Palynol.* **106,** 131–151 (1999).

Taberlet, P., Gielly, L. Patou, G. & Bouvet, J. Universal primers for amplification of three non-coding regions of chloroplast DNA. *Plant Mol. Biol.* **17,** 1105–1109 (1991).

Takahashi, M., Crane, P. R. & Ando, H. *Esgueiria futabensis* sp. nov., a new angiosperm flower from the Upper Cretaceous (Lower Coniacian) of northeastern Honshu, Japan. *Paleontological Research* **3,** 81–87 (1999).

Tewes, L. J., Michling, F., Koch, M. A. & Müller, C. Chemical diversity mirrors genetic diversity and might be a key advantage in intracontinental invasion of the perennial *Bunias orientalis. J. Ecol.*, DOI: 10.1111/1365-2745.12869*.*

Supplementary Material Figure 1

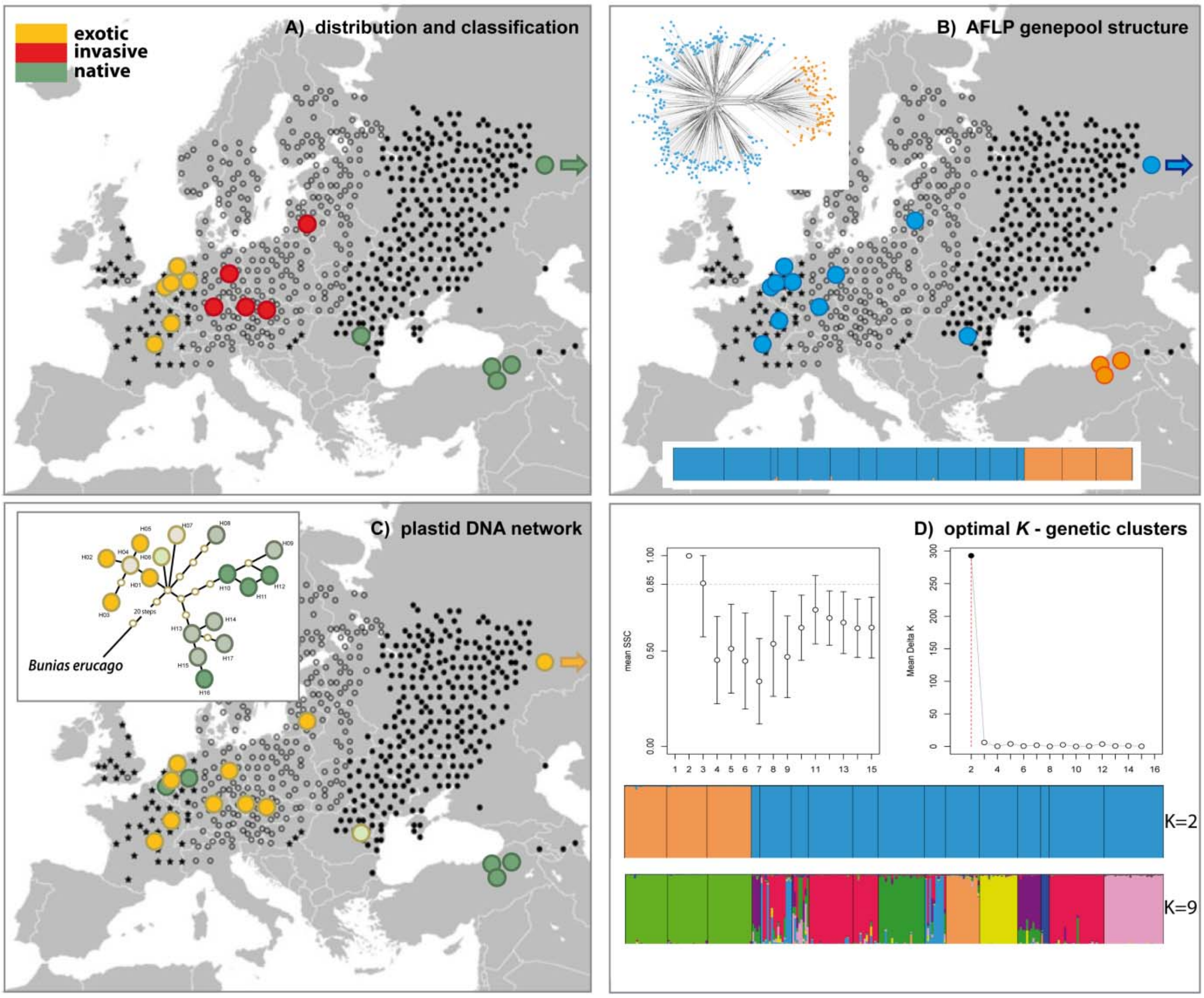

(A) Distribution of sampled populations for initial screening of genetic variation distribution within and between populations, (B) Results from STRUCTURE and Network analysis using optimal *K*=2, (C) Distribution of plastid haplotypes, (D) evaluation of optimal K and visualization for results of non-ideal *K*=9. Maps were generated in R using the mapdata package (A language and Environment for Statiscal Computing, R Core Team, R Foundation for Statiscal Computing, Vienna, Austria, 2017, https://www.R-project.org; mapdata: Extra Map Databases, R package version 2.2-6, https://CRAN.R-project.org/package=mapdata;), and figures were drawn in Inkscape v 0.91 (https://inkscape.org/). In A-C previous classification of the species distribution range [16] according to “native” (black dots), invasive (introduced during the last 250 years and invasive in this region; white dots) and exotic (recently introduced and not invasive, black stars) is indicated.

Supplementary Material Figure 2

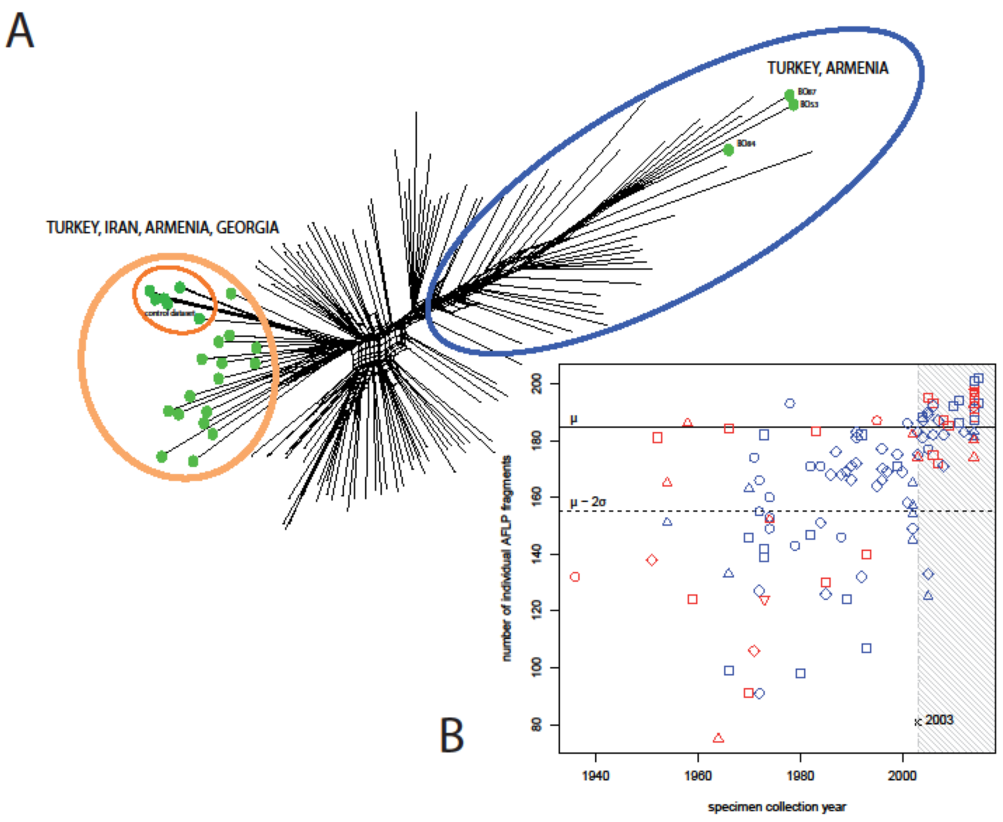

AFLP results for the entire data set prior final selection of accessions. (A) SplitsTree graph demonstrating unexpected grouping of some Turkisch/Armenian accessions and artificially long branches (blue circle), (B) Visualization of correlation between the age of the material and the number of AFLP loci scored. Colour code (blue/red) distinguishes different ALPF experiments; symbols indicate geographic origin: circle: America, triangle: Caucaus/Irano-Turanian, box: Europe, diamond: Scandinavia, inverse triangle: Central Asia.

Supplementary Material Figure 3

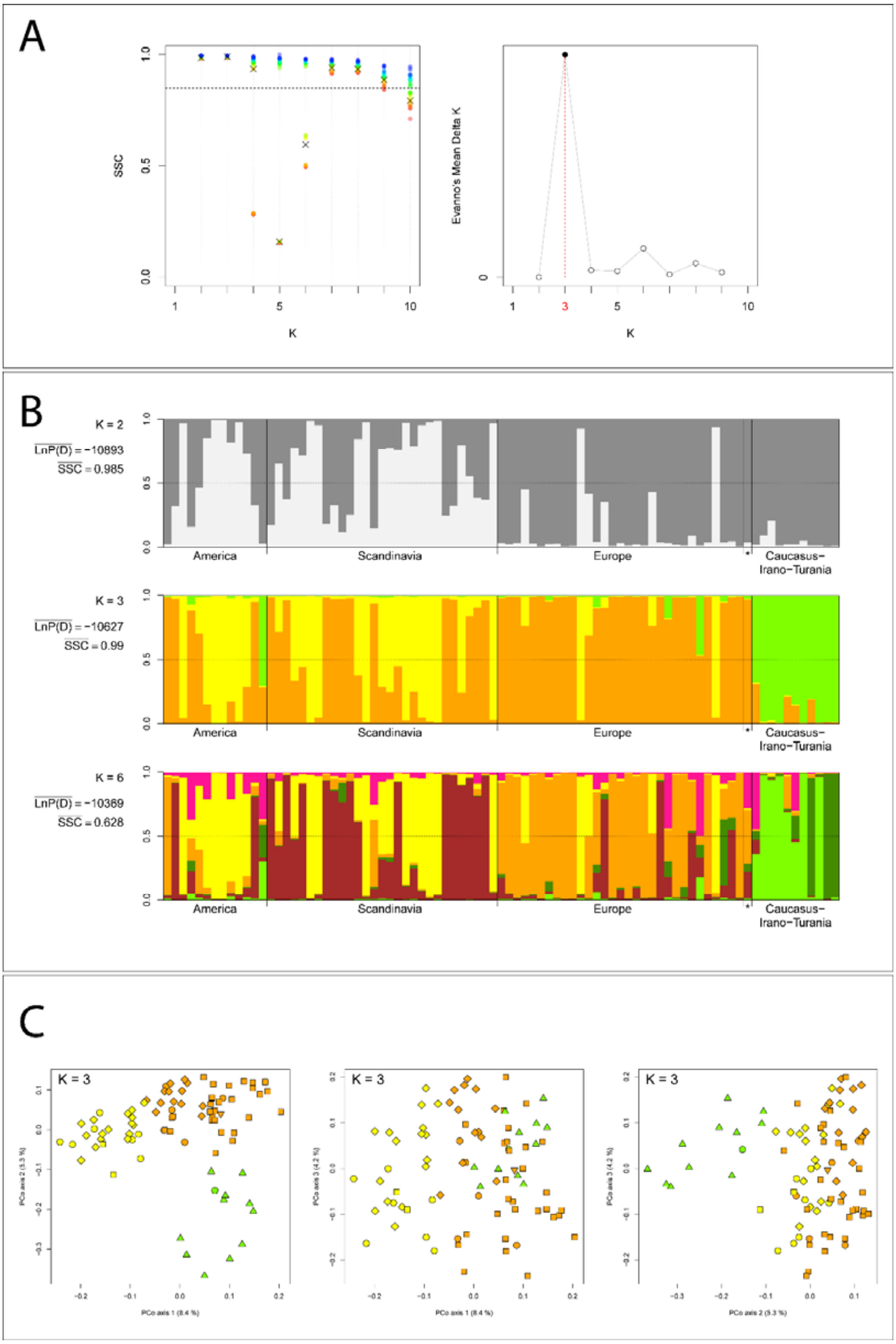

(A) Evaluation of optimal K for the large AFLP dataset and (B) corresponding STRUCTURE plots of genetic assignment using the admixture model with correlated allele frequencies. Asterik marks the position of the sample from Asia. Results of PCoA analysis are shown (C) for combinations of the first three coordinates. Symbols indicate geographic origin: circle: America, triangle: Caucaus-Irano-Turanian, box: Europe, diamond: Scandinavia, inverse triangle: Central Asia.

Supplementary Material Figure 4

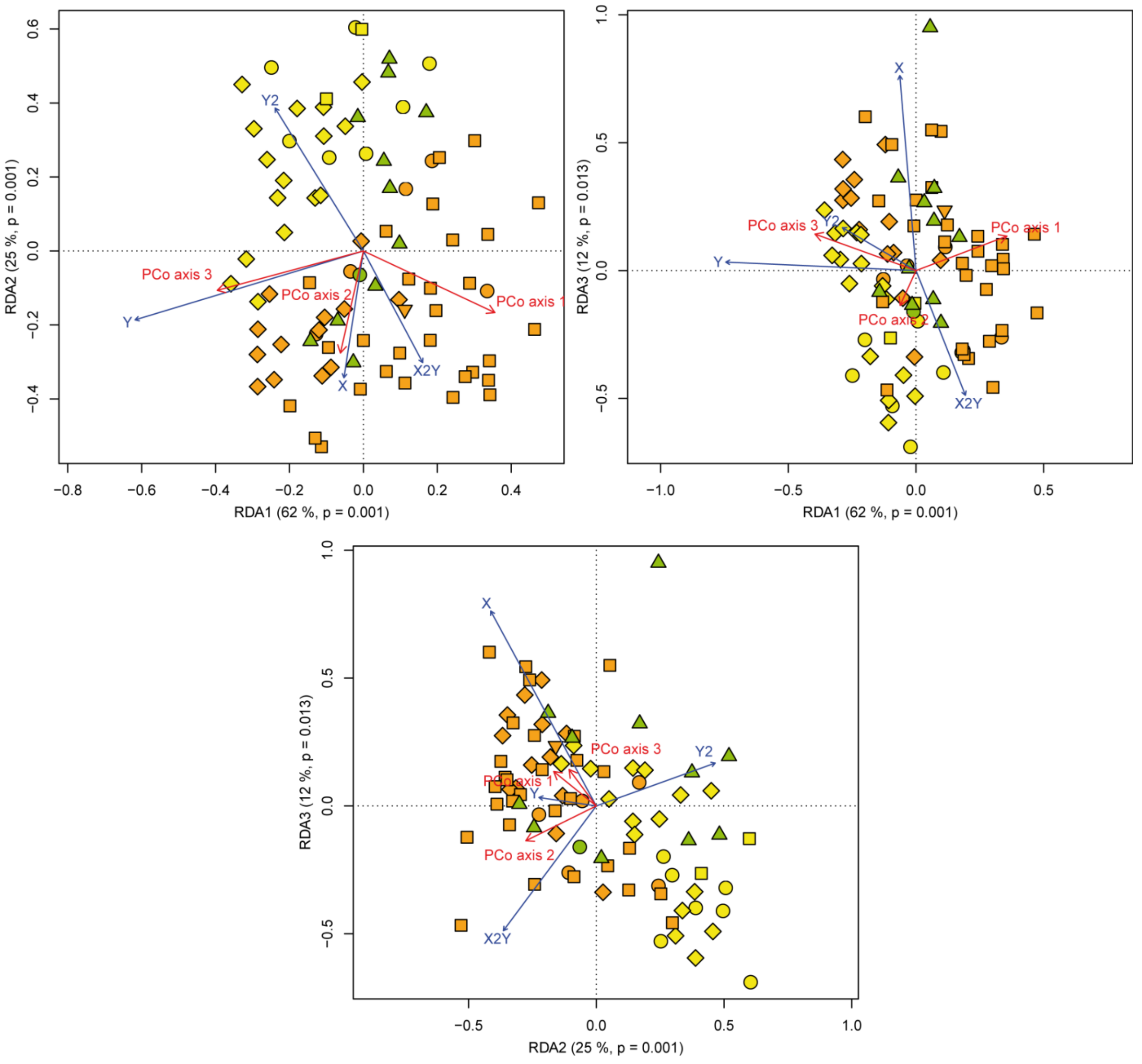

Results from (partial) redundancy analysis (RDA) on the data matrix comprising significant eigenvectors obtained by PCoA of the association matrix of genetic dissimilarities (AFLP dataset). The set of constraining variables comprised (in this order) nine 3rd degree orthogonal polynomials of geographic coordinates and BIOCLIM variables 10, 11, 18 and 19 at a spatial resolution of 10 arc minutes (mean temperature and precipitation of the warmest and coldest quarter respectively. After forward model selection, only four model terms (spatial terms x^2y, y, x and y^2) were retained and are indicated accordingly.

Suppl. Material Table 1

Information on the herbarium material of *Bunias orientalis* individuals used in this study. Geographic coordinates have been estimated from original sampling information. [Genbank codes for *trn*L intron, *trn*LF intergenic spacer and *trn*G intron are provided.]

LabID/Herbarium no./Genbank no. (*trn*L, *trn*LF, *trn*G)/Locality/geographic coordinates (longitude and latitude)/cpDNA haplotype/ collection year

BO001 H617327 KX720598 KX720748 KX720898 Finland Nâtö 60,04331 19,97421	H1	1985
BO002 H635321 KX720599 KX720749 KX720899 Finland Hamina 60,57057 27,19240	H2	1987
BO003 H666145 KX720600 KX720750 KX720900 Finland Ödkarby 60,30905 19,98001	H1	1991
BO004 H666252 KX720601 KX720751 KX720901 Finland Saltvik 60,27520 20,04798	H1	1991
BO005 H666269 KX720602 KX720752 KX720902 Finland Bomarsund 60,21666 20,23333	H1	1991
BO006 H729303 KX720603 KX720753 KX720903 Finland Suittila 60,33373 24,06841	H2	1990
BO007 H697855 KX720604 KX720754 KX720904 Finland Backas 60,27753 24,95595	H2	1995
BO008 H818117 KX720605 KX720755 KX720905 Finland Helsinki 60,14846 24,99102	H2	2006
BO009 H812686 KX720606 KX720756 KX720906 Finland Karkku 61,44577 23,04505	H2	2007
BO010 H827447 KX720607 KX720757 KX720907 Finland Hämeenlinna 61,00245 24,47835	H1	2012
BO011 H810637 KX720608 KX720758 KX720908 Finland Tykkimäki 60,88625 26,78998	H2	2005
BO012 H715473 KX720609 KX720759 KX720909 Finland Kristiinankaupunki 62,27449 21,38011	H2	1996
BO013 H812550 KX720610 KX720760 KX720910 Finland Lapävirta 62,48949 27,78751	H1	2007
BO014 H688346 KX720611 KX720761 KX720911 Finland Möhkö 62,64116 31,28919	H1	1992
BO015 H1670724 KX720612 KX720762 KX720912 Russia Gogland 60,05444 26,98500	H1	1992
BO016 H1683534 KX720613 KX720763 KX720913 Russia Salmiyarvi 69,43012 30,13192	H1	1996
BO017 H1683541 KX720614 KX720764 KX720914 Finland Jäniskoski 69,58130 28,70332	H2	1996
BO018 L1819221 KX720615 KX720765 KX720915 France Drusenheim 48,75717 7,96096	H1	1980
BO019 L1819224 KX720616 KX720766 KX720916 Czech Republic Bruno 49,09702 16,44533	H2	1973
BO020 L1819226 KX720617 KX720767 KX720917 France Bièvres 49,49287 3,71017	H5	1982
BO021 L1819236 KX720618 KX720768 KX720918 Germany Kleinsassn 50,55232 9,87420	H3	1966
BO022 U1164296 KX720619 KX720769 KX720919 Germany Kall 50,52447 6,55029	H1	1973
BO023 MA724280 KX720620 KX720770 KX720920 Norway Haslum 59,92194 10,57138	H1	2003
BO024 MA729553 KX720621 KX720771 KX720921 Estonia Tallinn 59,43118 24,75488	H1	2005
BO025 MA743179 KX720622 KX720772 KX720922 Armenia Sevan 40,59505 44,96636	H14	2005
BO026 MA763894 KX720623 KX720773 KX720923 Georgia Juta 42,56666 44,76666	H6	2002
BO027 WU066978 KX720624 KX720774 KX720924 Austria Lassee 48,21905 16,85645	H6	2011
BO028 WU062367 KX720625 KX720775 KX720925 Austria Traiskirchen 47,99496 16,29063	H3	2011
BO029 WU029946 KX720626 KX720776 KX720926 Austria Ebreichsdorf 47,97165 16,40928	H3	2004
BO030 WU081582 KX720627 KX720777 KX720927 Austria Wimpassing 47,91685 16,42711	H2	1999
BO031 WU081581 KX720628 KX720778 KX720928 Austria Vienna 48,16686 16,46987	H3	1992
BO032 N05/138-1110 KX720629 KX720779 KX720929 Sweden Klagshamn 55,53314 12,92017	H2	1984
BO033 N05/141-851 KX720630 KX720780 KX720930 Sweden Ljungby 56,63286 16,14370	H1	1986
BO034 N339-740 KX720631 KX720781 KX720931 Sweden Fridhem 59,18789 16,74064	H2	1990
BO035 N339-741 KX720632 KX720782 KX720932 Sweden Läggesta 59,24818 17,17855	H4	1988
BO036 S10-33201 KX720633 KX720783 KX720933 Sweden Stockholm 59,37569 18,04573	H1	2008
BO037 S-A0940-431 KX720634 KX720784 KX720934 Sweden Valbo 60,64303 17,00825	H1	2008
BO038 N08/63-10 KX720635 KX720785 KX720935 Sweden Torsåker 60,51044 16,47133	H2	2005
BO039 N04/122-7 KX720636 KX720786 KX720936 Sweden Hedesunda 60,39373 17,00649	H2	2002
BO040 N03/1-204 KX720637 KX720787 KX720937 Sweden Ovansjö 62,23333 17,38333	H1	2001
BO041 N475-440 KX720638 KX720788 KX720938 Sweden Högbo 58,03333 15,29999	H1	2000
BO042 N06/55-14 KX720639 KX720789 KX720939 Sweden Sundsvall 62,39081 17,30692	H1	2004
BO043 N04/51-26 KX720640 KX720790 KX720940 Sweden Grundsunda 63,36666 19,20000	H1	2003
BO044 N06/31-222 KX720641 KX720791 KX720941 Sweden Piteå 65,31669 21,48003	H1	2004
BO045 S-N0592-1127 KX720642 KX720792 KX720942 Sweden Östergarn 57,41833 18,87994	H2	1972
BO046 S15-25237 KX720643 KX720793 KX720943 Armenia Dilidschan 40,74083 44,86305	H14	2014
BO047 N464-981 KX720644 KX720794 KX720944 Sweden Mjölby 58,32269 15,13353	H1	2001
BO048 BC829998-2 KX720645 KX720795 KX720945 Germany Regensburg 49,03018 12,15868	H2	1993
BO049 BC901187 KX720646 KX720796 KX720946 Armenia Garni 40,11556 44,74259	H13	2002
BO050 BC900848-2 KX720647 KX720797 KX720947 Armenia Meghradzor 40,58710 44,62371	H13	2002
BO051 BC902207 KX720648 KX720798 KX720948 Armenia Yeghegis 39,87261 45,35751	H15	2002
BO052 BC901965 KX720649 KX720799 KX720949 Armenia Jermuk 39,82673 45,68973	H14	2002
BO054 BC900137-2 KX720650 KX720800 KX720950 Armenia Tsovagyugh 40,60326 44,96292	H13	2002
BO055 QFA612411 KX720651 KX720801 KX720951 Canada LaGrosse-Île 47,02846 -70,67137	H3	1971
BO056 QFA612409 KX720652 KX720802 KX720952 Canada Falardeau 48,62086 -71,11113	H2	1974
BO057 QFA612410 KX720653 KX720803 KX720953 Canada L'Islet 47,11447 -70,36485	H2	1978
BO058 QFA393066 KX720654 KX720804 KX720954 Canada St-Croix 46,62360 -71,73347	H2	1979
BO060 QFA588616 KX720655 KX720805 KX720955 Poland Lwówek Slaski 51,11128 15,58822	H2	2010
BO061 QFA505071 KX720656 KX720806 KX720956 Canada Chomedey 45,54509 -73,76237	H2	2006
BO062 QFA461450 KX720657 KX720807 KX720957 Canada Sainte-Rose 45,60918 -73,77775	H2	1988
BO063 QFA452771 KX720658 KX720808 KX720958 Norway Haslum 59,92936 10,56424	H1	1999
BO064 QFA524558 KX720659 KX720809 KX720959 Denmark Høm 55,40670 11,74635	H1	1972
BO065 QFA461451 KX720660 KX720810 KX720960 Czech Republic Moravský Krumlov 49,04893 16,31169	H2	1989
BO066 QFA514596 KX720661 KX720811 KX720961 Belgium Pesche 50,04230 4,45861	H3	2002

BO067 QFA418452 KX720662 KX720812 KX720962 Canada Pointeau Platon 46,66261 -71,83767	H2	1989
BO068 QFA496747 KX720663 KX720813 KX720963 Canada Montréal 45,57007 -73,59672	H2	2005
BO069 QFA118932 KX720664 KX720814 KX720964 Canada Grosse-Île 47,02728 -70,67054	H3	1971
BO070 QFA248755 KX720665 KX720815 KX720965 Czech Republic Bruno 49,19509 16,60673	H2	1973
BO071 QFA450315 KX720666 KX720816 KX720966 Canada Montréal 45,61716 -73,58487	H2	1984
BO072 QFA126536 KX720667 KX720817 KX720967 Canada Causapscal 48,36660 -67,24450	H1	1972
BO073 QFA166536 KX720668 KX720818 KX720968 Canada Falardeau 48,62086 -71,11113	H2	1974
BO074 QFA329522 KX720669 KX720819 KX720969 Canada Saint-Basile 46,74583 -71,81250	H2	1982
BO075 QFA166561 KX720670 KX720820 KX720970 Canada Falardeau 48,62086 -71,11113	H2	1974
BO076 QFA254169 KX720671 KX720821 KX720971 Canada Causapscal 48,36660 -67,24450	H1	1972
BO077 HEID503346 KX720672 KX720822 KX720972 Germany Irndorf 48,08129 8,98338	H3	2015
BO078 HEID503315 KX720673 KX720823 KX720973 Germany Mannheim 49,47495 8,51848	H6	2015
BO079 L1819229 KX720674 KX720824 KX720974 Romania Borzesti 46,23780 26,81805	H6	1970
BO080 HEID505063 KX720675 KX720825 KX720975 Germany Muggendorf 49,91869 11,30270	H2	1992
BO081 H720394 KX720676 KX720826 KX720976 Russia Yakkima 61,51712 30,13755	H1	1997
BO082 E00376425 KX720677 KX720827 KX720977 Turkey Kavussahapdagi Tepe 38,04999 42,95000	H15	1954
BO083 E00376427 KX720678 KX720828 KX720978 Turkey Kagizman 40,12429 43,17729	H15	1966
BO086 E00376434 KX720679 KX720829 KX720979 Turkey Piskasir 37,42102 44,14392	H15	1970
BO087 E00376438 KX720680 KX720830 KX720980 Turkey Ladik 40,91204 35,89482	H7	1964
BO089 E00748486 KX720681 KX720831 KX720981 Ukraine Chaikovs'ke 44,82915 34,32856	H2	1959
BO093 E00754529 KX720682 KX720832 KX720982 USA AnnArbor 42,28061 -83,72691	H16	1936
BO094 G00414672 KX720683 KX720833 KX720983 France Brénod 46,05979 5,60757	H3	1993
BO096 G00414670 KX720684 KX720834 KX720984 Switzerland Romainmôtier 46,69334 6,46139	H5	1985
BO097 G00414673B KX720685 KX720835 KX720985 France Mieussy 46,13393 6,52432	H1	1989
BO098 G00414669B KX720686 KX720836 KX720986 Switzerland Filet 46,36001 8,05169	H2	1984
BO101 G00414657 KX720687 KX720837 KX720987 Iran Qotur 38,47307 44,40776	H15	1974
BO102 G00414659 KX720688 KX720838 KX720988 Turkey Tamdere 40,50291 38,35638	H7	1958
BO103 G00414656 KX720689 KX720839 KX720989 Uzbekistan Burchmulla 41,60000 70,10000	H4	1973
BO104 G00414665 KX720690 KX720840 KX720990 France Saint-Auban 43,84847 6,72662	H3	2006
BO105 G00414664 KX720691 KX720841 KX720991 Poland Kostrze 50,01666 19,85000	H1	2007
BO107 G00414661 KX720692 KX720842 KX720992 Poland Zakopane 49,28857 19,94836	H5	1983
BO108 G00414660 KX720693 KX720843 KX720993 Russia Morozki 56,20740 37,52709	H2	1971
BO109 BM001191103 KX720694 KX720844 KX720994 Sweden Gryta 59,68969 17,44748	H1	1951
BO110 BM001191104 KX720695 KX720845 KX720995 Finland Tampere 61,47355 23,25318	H2	1959
BO111 BM001191106 KX720696 KX720846 KX720996 Luxembourg Reisdorf 49,86966 6,26789	H3	1952
BO112 BM001191107 KX720697 KX720847 KX720997 Romania Gheorgheni 46,78813 25,66928	H6	1970
BO114 BM001191100 KX720698 KX720848 KX720998 Turkey Kotum 38,44982 42,32276	H15	1954
BO117 QFA547949 KX720699 KX720849 KX720999 Belgium Gosselies 50,46897 4,43224	H2	1953
BO118 QFA125003 KX720700 KX720850 KX721000 Poland Slaska 51,20032 16,21191	H6	1966
BO120 HEID502915 KX720701 KX720851 KX721001 Armenia Yeritsvank 39,28222 46,48305	H15	2014
BO121 HEID400872 KX720702 KX720852 KX721002 Austria Wegscheid 47,68850 15,32643	H2	2015
BO122 HEID500569 KX720703 KX720853 KX721003 Germany Bochum 51,48116 7,21630	H3	2015
BO123 NCY008618 KX720704 KX720854 KX721004 France Villa des Fleurs 49,09260 5,41433	H2	2006
BO124 NCY012349 KX720705 KX720855 KX721005 France Laxou 48,69147 6,12837	H1	2008
BO125 NCY017586(HEID) KX720706 KX720856 KX721006 France Nancy 48,72472 6,18861	H1	2007
BO126 HEID405748 KX720707 KX720857 KX721007 Austria Eggenburg 48,64008 15,82075	H2	2009
BO127 HEID501057 KX720708 KX720858 KX721008 Armenia Pushkini Lerrnants'k' 40,91111 44,43194	H8	2003
BO128 V231347 KX720709 KX720859 KX721009 Canada Lytton 50,24000 -121,58027	H2	2003
BO129 V141657 KX720710 KX720860 KX721010 USA South Hadley 42,25400 -72,57624	H16	1972
BO130 V210551 KX720711 KX720861 KX721011 Canada Botanie Lake 50,33333 -121,55000	H2	1995
AL08 HEID s.n. KX720596 KX720746 KX720896 Russia Altai 50,819489 86,273566	H2	
AL12 HEID s.n. KX720597 KX720747 KX720897 Russia Altai 50,819489 86,273566	H2	
CZB15 HEID505134 KX720712 KX720862 KX721012 Czech Republic Zavadilka 49,942897 14,06791	H3	
CZB28 HEID505134 KX720713 KX720863 KX721013 Czech Republic Zavadilka 49,942897 14,06791	H3	
CZD18 HEID505172 KX720714 KX720864 KX721014 Czech Republic Drhovy 49,745753 14,227816	H3	
CZD21 HEID505172 KX720715 KX720865 KX721015 Czech Republic Drhovy 49,745753 14,227816	H3	
DI25 HEID505197 KX720716 KX720866 KX721016 Netherlands Driel 51,967834 5,852842	H6	
DI29 HEID505197 KX720717 KX720867 KX721017 Netherlands Driel 51,967834 5,852842	H6	
DR08 HEID505141 KX720718 KX720868 KX721018 Netherlands Drempt 52,006494 6,160406	H2	
DR19 HEID505141 KX720719 KX720869 KX721019 Netherlands Drempt 52,006494 6,160406	H2	
GO04 HEID s.n. KX720720 KX720870 KX721020 France Gondreville 48,68713 5,964921	H3	
GO20 HEID s.n. KX720721 KX720871 KX721021 France Gondreville 48,68713 5,964921	H3	
JE12 HEID505159 KX720722 KX720872 KX721022 Germany Jena 50,873581 11,595993	H1	
JE33 HEID505159 KX720723 KX720873 KX721023 Germany Jena 50,873581 11,595993	H1	
LT07 HEID505167 KX720724 KX720874 KX721024 Lithuania Vilnius 54,68083 25,345823	H2	
LT13 HEID505167 KX720725 KX720875 KX721025 Lithuania Vidukl˙e 55,415571 22,847756	H2	
OS09 HEID505132 KX720726 KX720876 KX721026 Germany Osnabrück 52,281087 8,028517	H16	
OS17 HEID505138 KX720727 KX720877 KX721027 Germany Osnabrück 52,281087 8,028517	H16	
OS28 HEID505138 KX720728 KX720878 KX721028 Germany Osnabrück 52,281087 8,028517	H16	
PA03 HEID s.n. KX720730 KX720880 KX721030 France Pasques 47,366359 4,855931	H2	
PA15 HEID s.n. KX720731 KX720881 KX721031 France Pasques 47,366359 4,855931	H2	
RO12 HEID505133 KX720732 KX720882 KX721032 Romania Iasi 47,1872439 27,5573941	H2	
RO20 HEID505133 KX720733 KX720883 KX721033 Romania Iasi 47,1872439 27,5573941	H6	
T417 HEID505003 KX720734 KX720884 KX721034 Turkey Rize 40,738746 40,735271	H9	
TA14 HEID505181 KX720735 KX720885 KX721035 Turkey Rize 40,738257 40,752539	H12	
TA17 HEID505181 KX720736 KX720886 KX721036 Turkey Rize 40,738257 40,752539	H12	
TA26 HEID505181 KX720737 KX720887 KX721037 Turkey Rize 40,738257 40,752539	H12	
TC10 HEID505202 KX720738 KX720888 KX721038 Turkey Rize 40,73288 40,790213	H11	

TC31	HEID505202	KX720739	KX720889	KX721039	Turkey	Rize	40,73288 40,790213	H12
TD23	HEID505192	KX720740	KX720890	KX721040	Turkey	Rize	40,738746 40,735271	H12
TD25	HEID505192	KX720741	KX720891	KX721041	Turkey	Rize	40,738746 40,735271	H12
WA08	HEID505149	KX720742	KX720892	KX721042	Netherlands	Wageningen	51,98097 5,673719	H10
WA11	HEID505149	KX720743	KX720893	KX721043	Netherlands	Wageningen	51,98097 5,673719	H10
WU11	HEID505175	KX720744	KX720894	KX721044	Germany	Würzburg	49,849161 9,865662	H2
WU12	HEID505175	KX720744	KX720894	KX721044	Germany	Würzburg	49,849161 9,865662	H5

Complete plastid genomes:

BO026	GCA_900231815
RO12	LN877378
BO118	GCA_900231825
T417	LN877379
OS09	LN877376
OSBU12478	LN877377

Suppl. Material Table 2

PCoA of 85 AFLP genotypes of Bunias orientalis. Eigenvalues and percent of total variation explained by corresponding PCo axis and corresponding values obtained by the broken-stick model (bsm) for axes 1 to 4. Sum of eigenvalues for all 84 PCo axes was 14.54469.

PCo axis	1	2	3	4
eigenvalue (PCoA)	1.220	0.777	0.607	0.455
eigenvalue (bsm)	0.059	0.047	0.041	0.038
percent of total variance	8.41	5.34	4.17	3.13
percent of total variance (bsm)	5.913	4.736	4.148	3.756
% PCoA > % bsm	Yes	yes	yes	no

Suppl. Material Table 3a

Summary statistics of AFLP data; n – number of samples; S – number of segregating sites; θ(S) Watterson's estimator of the expected number of sites; θ(π) observed, average number of segregating sites; D – Tajima's D; p(D) – p value for D obtained via simulations of 10,000 neutral genealogies.

population	N	S	θ (π)	θ (S)	D	p(D)
North America	13	141	48.41026	45.43681	0.29939	0.66130
Europe (complete)	60	199	48.42486	42.67452	0.47615	0.74270
+ Scandinavia	29	162	46.57635	41.25107	0.50073	0.75270
+ (Central) Europe	31	172	44.77419	43.05396	0.15352	0.62260
Caucasus-Irano-Turanian	11	146	47.81818	**49.84690**	-0.19596	0.45030

Suppl. Material Table 3b

AFLP Fragment summary table

		number of fragments							
					private fragments				
population	N[1]	total	fixed	polymorphic	fixed	polymorphic			
							shared[2]	unique[3]	rare (10%)[4]
North America	13	185	44	141	0	1	0	1	-
Europe (complete)	60	212	13	199	0	17	12	5	7
Scandinavia	29	187	25	162	0	2	1	1	0
(Central) Europe	31	207	35	172	0	10	6	4	2
Caucasus-Irano-Turanian	11	187	41	146	0	21	11	10	-
total	85	236	0	236	-	-	-	-	-

[1]sample size

[2]shared (polymorphic) private fragments are fragments that were observed multiple times in a single population

[3]unique, private fragments are fragments that were observed only once. Consequently they are private to a single population.

[4]rare, private fragments are fragments that were observed in less than 10% of samples from a single population. For populations Scandinavia, (Central) Europe and Europe (complete), 10% corresponded to 3, 3 and 6 samples respectively. Note that for populations North America and C-I-T, 10% corresponds to a single observation.

'-' indicates that a summary statistic did not apply and was not calculated; also see [2].
